# Lipid-Based Delivery Systems for Therapeutic Glycoproteins: Current Advances, Challenges, and Future Perspectives

**DOI:** 10.3390/pharmaceutics18091045

**Published:** 2026-08-22

**Authors:** Hamad Alrbyawi

**Affiliations:** Department of Pharmaceutics and Pharmaceutical Industries, College of Pharmacy, Taibah University, Al-Madinah Al-Munawarah 41477, Saudi Arabia; hrbyawi@taibahu.edu.sa

**Keywords:** therapeutic glycoproteins, lipid-based nanocarriers, targeted drug delivery, liposomes, solid lipid nanoparticles, glycoprotein delivery

## Abstract

Therapeutic glycoproteins, a pivotal class of biopharmaceuticals, have transformed modern medicine through their broad applications in oncology, immunotherapy, and infectious disease management. Their structural complexity and biological specificity make them highly effective in targeting disease pathways; however, challenges related to stability, bioavailability, and delivery efficacy limit their full potential. Recent advancements in delivery technologies have sought to address these challenges through innovative approaches such as nanotechnology-based carriers, controlled-release systems, and molecular engineering. These strategies have demonstrated the ability to enhance glycoprotein stability, optimize pharmacokinetics, and achieve targeted delivery with minimal off-target effects. This review provides a comprehensive overview of state-of-the-art lipid-based delivery systems specifically designed to overcome the unique pharmaceutical challenges associated with therapeutic glycoproteins, highlighting their design principles, formulation strategies, mechanisms of encapsulation and release, and therapeutic advantages in improving glycoprotein stability, bioavailability, targeted delivery, and treatment efficacy. In addition to surveying the current landscape, this review delves into the key challenges impeding the widespread adoption of advanced delivery systems, including immunogenicity, manufacturing scalability, and clinical translation. The review concludes with insights into emerging trends in the development of lipid-based delivery systems, positioning glycoprotein therapeutics at the forefront of innovation in biopharmaceuticals. This overview of advancements and challenges aims to provide a roadmap for future progress in the field of glycoprotein delivery and therapeutic applications.

## 1. Introduction

Therapeutic glycoproteins have become essential agents in biopharmaceutical innovation, revolutionizing modern medicine through their remarkable specificity and efficacy in addressing physiological and pathological conditions. Glycoproteins are complex biomolecules formed via glycosylation, a post-translational modification involving the covalent attachment of carbohydrate moieties (glycans) to a protein backbone. This modification is a highly regulated, enzyme-mediated process that occurs predominantly within the endoplasmic reticulum (ER) and Golgi apparatus, where sequential glycan assembly and processing confer precise structural features to the protein. Within the ER and Golgi apparatus, protein glycosylation predominantly includes N-glycosylation, O-glycosylation, C-mannosylation, and the biosynthesis of glycosylphosphatidylinositol (GPI)-anchored proteins, with N- and O-glycosylation being the most abundant [1]. N-glycosylation is the attachment of an oligosaccharide to the amide nitrogen of asparagine in a conserved sequence Asn-X-Ser/Thr motif, where X can be any amino acid except proline. This process starts in the ER upon addition of a pre-assembled oligosaccharide precursor and then is extensively trimmed and remodeled in the Golgi. N-linked glycans are key for protein folding, quality control, stability, and intracellular transport [2]. In contrast, O-glycosylation occurs through the stepwise addition of monosaccharides to the hydroxyl groups of serine or threonine residues, primarily within the Golgi apparatus. Unlike N-glycosylation, O-glycosylation does not rely on a defined consensus sequence or pre-assembled glycan precursor, resulting in greater structural heterogeneity. O-linked glycans are particularly significant in modulating protein–protein interactions, protease resistance, and mucosal barrier function [3]. Glycosylation is also a key regulator of glycoprotein structure and function, affecting protein stability, folding, receptor binding, and immunogenicity. This structural complexity provides glycoproteins with remarkable versatility, enabling them to participate in various biological processes such as cell signaling, immune regulation, and molecular recognition [4].

Glycoproteins exhibit a unique ability to perform highly specific biological functions, a property that arises from the precise combination of their protein backbone and covalently attached carbohydrate chains. While the amino acid sequence determines the core structure and catalytic or binding domains, the glycan moieties are crucial for fine-tuning its biological activity and specificity [5]. Glycoproteins’ broad biological functionality enables them to play crucial roles in essential processes, including cell-to-cell communication, modulation of immune responses, and regulation of enzymatic activities. They play essential roles in cell–cell recognition, intercellular communication, and signal transduction by acting as receptors, ligands, and adhesion molecules on cell surfaces [6]. In the immune system, glycoproteins regulate antigen presentation, immune cell activation, and host–pathogen interactions [7]. In enzymatic systems, glycosylation can influence catalytic activity and substrate specificity [8]. Furthermore, glycoproteins are essential components of extracellular matrices, mucosal barriers, and transport systems, emphasizing their importance in maintaining cellular integrity, homeostasis, and adaptive biological responses [9]. These attributes have driven the extensive use of glycoproteins in the development of therapeutic interventions targeting some of the most challenging illnesses. They have significantly reshaped treatment paradigms in oncology, infectious, autoimmune, and lysosomal storage diseases. For example, monoclonal antibodies, a prominent class of glycoproteins, have altered cancer therapy by providing highly precise targeting of tumor-specific antigens. The high specificity and efficacy of glycoproteins in various therapeutic applications result from their selective interactions with molecular targets, which limit adverse effects and improve clinical performance. This precision has transformed treatment strategies and enabled more personalized therapeutic interventions [10]. By exploiting their inherent structural complexity and broad biological functionality, glycoproteins continue to establish new standards in biopharmaceutical innovation, offering promising opportunities for the treatment and management of some of the most critical medical challenges.

Despite their therapeutic potential, glycoproteins present several challenges that limit their clinical application. Their inherent structural complexity, characterized by complex three-dimensional conformations and heterogeneous glycosylation profiles, poses significant difficulties for synthesis, purification, characterization, and scalable manufacturing [11]. Moreover, many glycoproteins display short biological half-lives as they undergo rapid clearance from the circulation through enzymatic degradation, renal elimination, and immune-mediated clearance. Consequently, frequent dosing regimens are required, which can negatively impact patient compliance and increase treatment-related costs [12]. Significant advancements have been achieved in overcoming these limitations through the development of innovative drug delivery platforms. Drug delivery systems such as lipid nanoparticles, polymeric carriers, hydrogels, receptor-targeted systems, and antibody–drug conjugates have demonstrated the ability to enhance glycoprotein stability, facilitate targeted delivery, improve pharmacokinetic profiles, and enable controlled release. These advances ultimately improve therapeutic efficacy while minimizing systemic toxicity and off-target effects.

Although several review articles have discussed protein therapeutics, biologics, or lipid-based nanocarriers independently [13,14,15], this review provides a comprehensive and focused analysis of lipid-based nanocarriers, including liposomes, lipid nanoparticles, solid lipid nanoparticles, nanostructured lipid carriers, lipid–polymer hybrid nanoparticles, and emerging lipid delivery platforms, for glycoprotein, used either as therapeutic cargoes or as targeting ligands and other functional components. Beyond summarizing recent advances, this review critically discusses the mechanistic roles of lipid composition, formulation design, surface engineering, and targeting strategies in overcoming glycoprotein-specific barriers such as enzymatic degradation, structural instability, immune recognition, limited bioavailability, and intracellular delivery. Furthermore, the review integrates current translational challenges, including manufacturing scalability, regulatory considerations, and future perspectives for the rational development of next-generation glycoprotein therapeutics.

## 2. Therapeutic Potential of Glycoproteins

Glycoproteins have exceptional therapeutic potential due to their structural complexity, biological specificity, and ability to interact selectively with molecular targets. The therapeutic applications of glycoproteins extend across a broad spectrum of medical fields such as infectious diseases, autoimmune disorders, and oncology, making them one of the most influential classes of biopharmaceutical agents (Table 1).

This table provides selected examples of approved therapeutic glycoproteins and is not intended to be exhaustive. Data were last accessed on July 2026. For updated information on approved indications and clinical use, readers should refer to the FDA database and official prescribing information. The therapeutic uses listed are representative and do not encompass all approved indications for each product.

**Figure 1:**
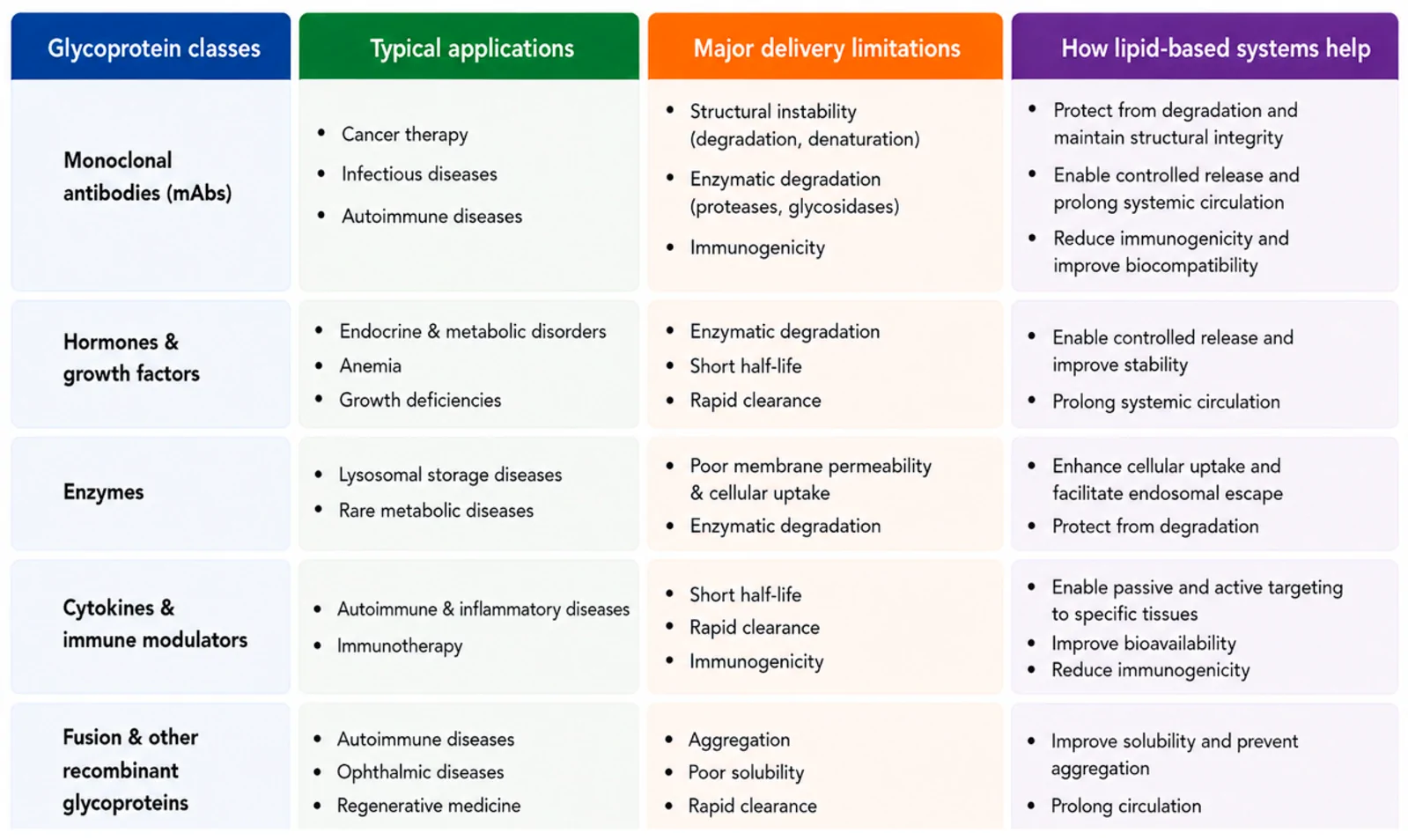
Conceptual relationship between therapeutic glycoprotein classes, their clinical applications, major delivery limitations, and the advantages of lipid-based delivery systems.

Monoclonal antibodies, a major class of glycoproteins, have revolutionized cancer treatment through their ability to highly selectively recognize tumor-associated antigens. Biologic agents such as trastuzumab, which targets human epidermal growth factor receptor 2 (HER2) in breast cancer, and rituximab, directed against CD20 protein in B-cell lymphomas, demonstrate the ability of glycoproteins to preferentially act on diseased tissues while limiting off-target interactions, thus reducing toxicity and improving clinical outcomes [16]. Alongside their established applications in oncology, glycoproteins have demonstrated significant therapeutic value in autoimmune disorders. Cytokine-based glycoproteins, such as interleukin-2 and interferon-beta, are widely used to modulate immune function in conditions including rheumatoid arthritis and multiple sclerosis [17]. Glycoproteins play critical roles in the prevention, diagnosis, and treatment of various infectious diseases due to their capability to mediate specific molecular recognition events between pathogens and host cells. Many viral surface antigens, including envelope glycoproteins, are the main targets for immune recognition and form the basis of modern vaccine design. The COVID-19 vaccines provide an impactful example of the use of glycoproteins in infectious disease control. The primary target of most COVID-19 vaccines is the SARS-CoV-2 spike (S) glycoprotein, a surface protein that enables viral entry into host cells by binding to the ACE2 receptor [18]. Moreover, enzyme replacement therapies for lysosomal storage disorders, such as those targeting mannose-6-phosphate receptors, demonstrate the clinical success of glycoprotein-based treatments [19]. Despite challenges related to stability, immunogenicity, and large-scale manufacturing, advances in bioengineering, glycoengineering, and drug delivery technologies have considerably expanded the therapeutic use of glycoproteins (Figure 1). Continued innovation in formulation, delivery, and molecular design is expected to further unlock the clinical potential of glycoprotein therapeutics.

## 3. Challenges in Glycoprotein Delivery

### 3.1. Stability and Degradation

Structural instability challenges commonly exhibited by glycoprotein molecules continue to pose significant limitations to their pharmaceutical applications, as these instabilities can adversely affect their overall therapeutic effectiveness. The macromolecular nature of glycoproteins, with their structural complexity and diversity, has significantly hindered the advancement of predictive approaches and universal strategies for both their chemical and physical stabilization.

Glycoproteins face similar chemical instability challenges as traditional small-molecule therapeutics, including acid–base reactions, redox processes, and chemical fragmentation. Additionally, their noncovalent nature makes them prone to physical instability issues, such as irreversible conformational changes and both local and global unfolding [20]. Furthermore, due to their colloidal nature, glycoproteins are susceptible to precipitation, surface adsorption, and nonnative supramolecular aggregation, pH, temperature, and concentration-dependent precipitation [21]. Glycoproteins are primarily administered intravenously rather than orally due to their susceptibility to chemical degradation by digestive system proteases. However, systemic proteolytic enzymes also make glycoproteins delivered via alternative routes vulnerable to proteolytic degradation. Consequently, the molecular stability of glycoproteins against proteolysis is closely tied to their in vivo therapeutic efficacy [22]. Therefore, the development of advanced delivery platforms capable of protecting glycoproteins from degradation and preserving their structural integrity has become a central focus in therapeutic glycoprotein research.

#### 3.1.1. Chemical Stability

Chemical stability represents a major challenge in glycoprotein delivery due to their vulnerability to various degradation pathways, which compromise their structural integrity, functionality, and therapeutic efficacy (Table 2). Reactive oxygen species generated during manufacturing, storage, or administration preferentially oxidize susceptible amino acid residues, including methionine, cysteine, tryptophan, tyrosine, and histidine, resulting in conformational alterations, aggregation, fragmentation, and reduced biological activity [23]. Oxidative damage may also increase immunogenicity by exposing cryptic epitopes or promoting aggregate formation [24]. In lipid-based delivery systems, oxidative degradation can be further exacerbated by lipid peroxidation, particularly when formulations contain unsaturated phospholipids or peroxide-containing excipients. Therefore, formulation strategies such as antioxidant incorporation, metal chelation, oxygen exclusion, light protection, and the use of low-peroxide excipients are essential to preserve glycoprotein integrity and maintain long-term product stability.

Deamidation is a common non-enzymatic degradation pathway that significantly affects the structural integrity and therapeutic performance of glycoprotein drugs. The reaction primarily involves the conversion of asparagine residues to aspartic acid or isoaspartic acid through a succinimide intermediate, resulting in changes to the protein backbone that can disrupt native conformation. These modifications may reduce biological activity, promote aggregation, alter pharmacokinetic behavior, and increase immunogenicity [25]. The extent of deamidation is influenced by amino acid sequence, protein conformation, pH, temperature, and storage conditions, with neutral to alkaline environments generally accelerating the reaction. Although lipid-based delivery systems can provide a protective microenvironment that limits conformational instability and exposure to external stressors, careful optimization of formulation parameters, including pH, excipient selection, and storage conditions, remains essential to minimize deamidation and maintain the stability and efficacy of therapeutic glycoproteins.

Hydrolysis, particularly under extreme pH conditions, can fragment proteins and degrade carbohydrate chains, damaging their structural and functional roles [26]. Hydrolytic reactions can cleave peptide bonds within the protein backbone, induce fragmentation, and disrupt glycosidic linkages, leading to structural destabilization and loss of biological activity. Hydrolysis may also alter glycosylation patterns, thereby influencing protein folding, receptor interactions, pharmacokinetics, and immunogenicity [27]. Encapsulation of therapeutic glycoproteins within lipid-based delivery systems can mitigate hydrolytic degradation by shielding proteins from aqueous and enzymatic environments while maintaining their native conformation. However, comprehensive optimization of formulation parameters, including lipid composition, internal pH, residual moisture, and storage conditions, remains essential to ensure long-term stability and therapeutic efficacy.

Non-enzymatic glycation, commonly referred to as the Maillard reaction, is an important chemical degradation pathway that can adversely affect the stability and therapeutic performance of glycoprotein drugs. The reaction involves the spontaneous interaction of reducing sugars with free amino groups of lysine residues or the protein N-terminus, leading to the sequential formation of Schiff bases, Amadori products, and advanced glycation end products. These modifications can alter protein conformation, reduce biological activity, promote aggregation, increase molecular heterogeneity, and enhance immunogenicity [28]. Disulfide bond scrambling or reshuffling during stress conditions can result in misfolding and aggregation, while isomerization of residues like aspartic acid may diminish receptor- binding affinity and therapeutic potency [29].

Aggregation constitutes one of the most significant challenges in glycoprotein formulation. Partial unfolding exposes hydrophobic domains that promote intermolecular interactions and formation of soluble oligomers or insoluble aggregates. Besides decreasing the concentration of active therapeutic molecules, aggregated proteins are more readily recognized by antigen-presenting cells and can stimulate anti-drug antibody formation, thereby reducing therapeutic efficacy and increasing the risk of adverse immune reactions [30].

In addition to molecular instability, poor membrane permeability severely restricts glycoprotein delivery. Their large molecular size, hydrophilic character, and limited lipophilicity prevent passive diffusion across epithelial and endothelial membranes, including the intestinal epithelium, pulmonary barrier, blood–brain barrier, and cellular plasma membrane [31]. Even after cellular uptake through endocytosis, many glycoproteins become trapped within endosomes and are subsequently degraded following lysosomal trafficking, further reducing intracellular bioavailability [32].

Collectively, these physicochemical limitations emphasize that successful glycoprotein delivery requires integrated formulation strategies capable of simultaneously preserving structural integrity, preventing enzymatic degradation and aggregation, enhancing membrane transport, and improving intracellular bioavailability. Lipid-based nanocarriers have emerged as promising platforms to address many of these challenges through molecular encapsulation, membrane fusion, surface engineering, and controlled intracellular release.

#### 3.1.2. Physical Instability

Physical stability challenges in glycoprotein delivery arise from their complex macromolecular structure and sensitivity to environmental factors, which can lead to structural modifications, loss of functionality, and reduced therapeutic efficacy (Table 3). Glycoproteins are susceptible to irreversible conformational changes due to environmental stresses such as variations in temperature, pH, or ionic strength, resulting in denaturation and impaired biological activity [33]. Their noncovalent interaction makes them susceptible to local and global unfolding, exposing hydrophobic regions that destabilize the molecule and promote aggregation or degradation. Precipitation under unfavorable conditions, including extreme pH or high temperature, further reduces bioavailability and complicates formulation [34]. In addition, protein precipitation can, compromise dose uniformity, shorten product shelf life, and increase immunogenicity due to the formation of particulate matter [35].

Surface adsorption is a major source of physical instability in therapeutic glycoproteins, arising from the interaction of protein molecules with solid–liquid and air–liquid interfaces encountered during manufacturing, storage, and administration [36]. Adsorption is mediated by hydrophobic, electrostatic, and hydrogen-bonding interactions that can induce conformational changes, leading to partial unfolding, aggregation, and irreversible loss of biological activity. The resulting reduction in protein concentration, together with the formation of surface-induced aggregates, may compromise therapeutic efficacy and increase immunogenicity. Factors such as low protein concentration, mechanical agitation, high surface-to-volume ratios, and prolonged storage further exacerbate adsorption. Additionally, glycoproteins are vulnerable to shear and mechanical stress during manufacturing, processing, storage, and transportation, leading to fragmentation, unfolding, or aggregation, all of which present significant obstacles in maintaining stability, therapeutic performance, and shelf life [37].

To minimize the physical instability, formulation strategies focus on optimizing pH and buffer composition, controlling protein concentration, incorporating stabilizing excipients, and maintaining appropriate storage conditions [38]. Lipid-based delivery systems provide an additional level of protection by encapsulating glycoproteins within a stabilizing lipid matrix, reducing exposure to environmental stressors and preserving protein solubility, thereby improving formulation stability and therapeutic performance.

#### 3.1.3. Enzymatic Instability

Glycoproteins are highly susceptible to enzymatic degradation, which compromises their stability, bioavailability, and therapeutic efficacy (Table 4). Proteases, such as endopeptidases and exopeptidases, hydrolyze peptide bonds within the protein backbone by, leading to structural instability, reduced biological activity, and rapid clearance [39]. In addition, glycosidases, including exoglycosidases and endoglycosidases, degrade the glycan moieties by hydrolyzing glycosidic bonds, removing glycan-mediated protection and changing pharmacokinetics and receptor interactions [40]. In some cases, proteases and glycosidases act synergistically, accelerating degradation and diminishing functional activity. This dual susceptibility to enzymatic attack highlights the challenges in maintaining the stability and efficacy of glycoprotein-based therapeutics during delivery and in vivo administration [41].

Enzymatic degradation is especially problematic for non-invasive routes of administration, such as oral and pulmonary delivery, where proteins are exposed to abundant digestive and mucosal enzymes. Lipid-based delivery systems provide an effective strategy for mitigating enzymatic instability by encapsulating glycoproteins within protective lipid matrices that limit enzyme accessibility, preserve structural integrity, and enhance bioavailability. Surface modification with polyethylene glycol (PEG) or targeting ligands can further enhance protection by reducing opsonization, minimizing enzymatic exposure, and promoting selective tissue accumulation [42].

### 3.2. Immunogenicity

Immunogenicity refers to the ability of a substance, such as a glycoprotein, to elicit an immune response in the body. Glycoproteins are inherently immunogenic due to their structural complexity stemming from the combination of protein and carbohydrate components. This immune detection may lead to the production of anti-drug antibodies, which can decrease therapeutic efficacy, modify pharmacokinetics, or trigger adverse immune reactions [43]. Immunogenicity of glycoproteins is influenced by various factors related to their protein and carbohydrate components, as well as their formulation and delivery (Table 5). The protein backbone contains peptide epitopes that can be recognized by T and B cells, triggering immune responses, while improper folding or aggregation may expose cryptic epitopes, provoking immunogenicity [44]. Additionally, glycoproteins derived from non-human sources are more likely to be perceived as foreign by the immune system [45]. The carbohydrate component also plays a crucial role as unique glycan structures, especially non-native moieties like α-Gal or Neu5Gc, can act as antigens that lead to immune recognition [46]. Mannose-rich glycans, in particular, can activate innate immune pathways via mannose receptors [47]. Aggregation of glycoproteins further enhances immune recognition, and impurities from production processes, such as host cell proteins or DNA, can provoke immune reactions. Moreover, the route of administration impacts immunogenicity, with subcutaneous or intramuscular routes inducing stronger immune responses due to extended exposure of the glycoprotein to antigen-presenting cells within lymphatic and peripheral tissues [48]. In contrast, intravenous administration usually results in rapid systemic distribution, which may reduce localized immune activation.

In addition to the intrinsic immunogenicity of therapeutic glycoproteins, repeated administration of glycoprotein therapeutics can induce the formation of anti-drug antibodies (ADAs), which may neutralize therapeutic activity, alter pharmacokinetics, and reduce clinical efficacy [49]. Furthermore, certain lipid nanoparticle formulations may activate the complement system through the binding of naturally occurring or pre-existing IgG and/or IgM antibodies to phospholipid head groups, resulting in complement activation-related pseudoallergy (CARPA), an acute infusion-related hypersensitivity reaction [50]. PEGylated lipid nanoparticles, while widely employed to prolong systemic circulation and reduce opsonization, have also been associated with the production of anti-PEG antibodies, potentially leading to accelerated blood clearance (ABC) upon repeated dosing and diminished therapeutic performance [51]. To minimize immune recognition, several formulation strategies have been explored, including optimization of lipid composition, reduction or replacement of PEG with alternative stealth polymers, incorporation of biomimetic or zwitterionic surface coatings, and careful control of particle size, surface charge, and ligand density. These approaches aim to improve long-term safety while preserving the pharmacokinetic and targeting advantages of lipid-based glycoprotein delivery systems.

### 3.3. Bioavailability

The bioavailability of glycoprotein-based drugs is strongly influenced by their large molecular size, structural complexity, route of administration, and susceptibility to enzymatic degradation. Intravenous (IV) administration ensures 100% bioavailability by delivering the drug directly into systemic circulation, whereas oral delivery suffers from poor bioavailability due to enzymatic degradation by proteases and glycosidases and limited absorption across the intestinal epithelium [52]. Subcutaneous or intramuscular routes offer moderate bioavailability but may face several challenges, such as slow absorption, immunogenicity, and enzymatic degradation. Enzymatic degradation considerably impacts glycoprotein activity, as proteolytic enzymes degrade their protein backbone and glycosidases remove carbohydrate chains, affecting both stability and receptor binding [53]. Additionally, the large molecular weight and structural complexity of glycoproteins hinder their diffusion across biological barriers, including epithelial layers and the blood–brain barrier [54]. Additional limitations to glycoprotein bioavailability arise from clearance mechanisms, including renal filtration of low-molecular-weight glycoproteins (below 60 kDa) and hepatic metabolism of larger species, alongside immune system recognition that may further accelerate the removal of glycoprotein therapeutics from circulation [55].

Glycoproteins often undergo fast clearance due to abnormal glycosylation and asialylation. Non-human or improperly processed glycans are recognized by the immune system or hepatic receptors, accelerating their removal. For example, high-mannose glycans are quickly cleared by mannose receptors on macrophages and hepatocytes [56]. Additionally, the loss of terminal sialic acids, a process known as asialylation, exposes galactose residues that are identified and targeted for clearance by the asialoglycoprotein receptor in the liver [57]. Furthermore, glycoproteins with Fc domains, such as monoclonal antibodies, may interact with Fc receptors on immune cells, accelerating their clearance [58]. Collectively, these factors profoundly influence the pharmacokinetic behavior, bioavailability, and therapeutic performance of glycoprotein-based therapeutics.

### 3.4. Production and Scalability

The production of glycoproteins, whether for therapeutic use or research purposes, involves complex biotechnological processes that face numerous challenges in achieving scalability and consistency. These challenges arise due to the complex structure of glycoproteins, the need for precise post-translational modifications, host cell limitations and the limitations of current production systems [59]. Achieving consistent glycan patterns is critical for maintaining their functionality and variability in glycosylation can decrease efficacy and increase immunogenicity. Common production systems, such as mammalian cells, are expensive and often yield low product levels, whereas microbial and plant systems often produce non-human glycan structures and require advanced engineering [60]. Scaling up production in bioreactors involves significant capital and operational expenses, with risks of contamination and inconsistent product quality [61]. Additionally, Downstream processing further complicates large-scale production. Glycoproteins are sensitive to shear stress, temperature fluctuations and pH changes, making purification processes such as chromatography and filtration technically demanding [62]. Addressing these challenges requires innovations in glycoengineering, bioreactor design, and efficient downstream processes to enable scalable and cost-effective production.

Scalable production technologies must ensure consistent particle size, encapsulation efficiency, and product quality while preserving the structural integrity and biological activity of sensitive glycoproteins. Recent advances in microfluidic manufacturing have enabled continuous, highly reproducible nanoparticle production with improved control over critical quality attributes compared with conventional batch methods [63]. Furthermore, implementation of Quality-by-Design (QbD) principles and Process Analytical Technology (PAT) facilitates systematic optimization, real-time process monitoring, and robust process control, thereby enhancing manufacturing consistency and regulatory compliance [64]. Additional challenges include the development of effective sterilization methods that do not compromise glycoprotein stability, maintenance of long-term storage stability through optimized formulations or lyophilization, and reliable cold-chain logistics required for temperature-sensitive products [65].

For lipid-based formulations, regulatory agencies additionally expect detailed characterization of critical quality attributes such as particle size distribution, polydispersity, zeta potential, encapsulation efficiency, lipid composition, release characteristics, and long-term stability. The development of biosimilar glycoproteins introduces further regulatory challenges, as demonstration of analytical, functional, and clinical comparability must also account for potential differences arising from complex lipid delivery systems. Regulatory expectations are increasingly guided by the International Council for Harmonization (ICH) guidelines, including quality risk management, pharmaceutical development, and lifecycle management, together with product-specific guidance issued by agencies such as the U.S. Food and Drug Administration (FDA). Guidelines such as ICH Q8 (Pharmaceutical Development) encourage a science- and risk-based approach to formulation design by identifying critical quality attributes (CQAs) and critical process parameters (CPPs) that influence product performance. ICH Q9 (Quality Risk Management) provides a systematic framework for identifying, evaluating, and controlling risks throughout product development and manufacturing, while ICH Q10 (Pharmaceutical Quality System) emphasizes lifecycle management and continual process improvement to ensure consistent product quality [66]. The application of these guidelines, together with current regulatory expectations from agencies, such as the FDA, supports robust manufacturing, product consistency, and successful clinical translation of lipid-based glycoprotein formulations.

The successful development of lipid-based nanocarriers requires careful control of both formulation characteristics and manufacturing parameters to ensure product quality, stability, and reproducibility. Although the specific critical quality attributes (CQAs), critical material attributes (CMAs), and critical process parameters (CPPs) differ among lipid-based nanocarrier systems, careful control of these factors is essential to ensure product quality, stability, manufacturability, and scalability [67]. Table 6 summarizes the principal CQAs, CMAs, CPPs, commonly used characterization methods, and major formulation challenges associated with the major lipid-based nanocarrier systems discussed in this review, providing a practical overview for formulation development and scale-up.

Within a QbD framework, successful development of lipid-based nanocarriers requires systematic identification and control of CQAs, CMAs, and CPPs. CQAs, including particle size, polydispersity index, zeta potential, encapsulation efficiency, drug loading, and release characteristics, directly influence the stability, safety, and therapeutic performance of the final formulation [68]. CMAs, such as lipid composition, lipid purity, cholesterol content, and physicochemical properties of the starting materials, govern nanoparticle formation, drug encapsulation, membrane integrity, and long-term storage stability. CPPs, including mixing conditions, homogenization pressure, extrusion parameters, flow rate, and cooling conditions, determine the reproducibility of the manufacturing process and significantly affect the resulting CQAs. Consequently, optimization of CMAs and CPPs is essential to achieve formulations with consistent CQAs, thereby minimizing aggregation, drug leakage, chemical degradation, and batch-to-batch variability while ensuring robust manufacturing and successful scale-up [69].

## 4. Lipid-Based Nanocarrier Systems

Nanoparticles (1–1000 nm) have emerged as advanced drug delivery systems based on the principle of encapsulating, adsorbing, or conjugating therapeutic agents to improve their stability, solubility, and bioavailability. These systems enable controlled and sustained drug release, reducing dosing frequency and maintaining optimal drug concentrations over time [70]. Furthermore, nanoparticles facilitate targeted delivery through passive mechanisms, such as the enhanced permeability and retention effect, or active targeting via surface modification with ligands that bind specific cellular receptors [71]. The materials used in nanoparticle formulation include biodegradable polymers (e.g., PLGA, chitosan), lipid-based systems (e.g., liposomes, solid lipid nanoparticles), inorganic materials (e.g., gold and silica nanoparticles), and protein-based carriers (e.g., albumin and gelatin) [72]. The selection of material influences key properties such as biocompatibility, drug loading capacity, release kinetics, and targeting efficiency.

Lipid-based delivery systems have demonstrated broad therapeutic potential across multiple disease areas, including cancer, autoimmune diseases, infectious diseases, metabolic disorders, and rare genetic disorders. Although the formulation principles are generally similar, the design requirements vary according to the underlying disease pathology and therapeutic objectives. In oncology, lipid nanocarriers are primarily engineered to improve tumor accumulation, prolong systemic circulation, and reduce off-target toxicity through passive and active targeting strategies [73]. In autoimmune and inflammatory diseases, they facilitate localized or sustained delivery of monoclonal antibodies and immunomodulatory glycoproteins, thereby reducing systemic immunosuppression and dosing frequency [74]. For infectious diseases, lipid-based systems protect glycoproteins from degradation while enhancing antigen presentation, mucosal delivery, or antibody-mediated neutralization [75]. In metabolic and rare diseases, particularly lysosomal storage disorders, lipid nanocarriers improve enzyme stability, prolong circulation, and promote receptor-mediated cellular uptake, thereby enhancing intracellular delivery and therapeutic efficacy [76].

**Figure 2:**
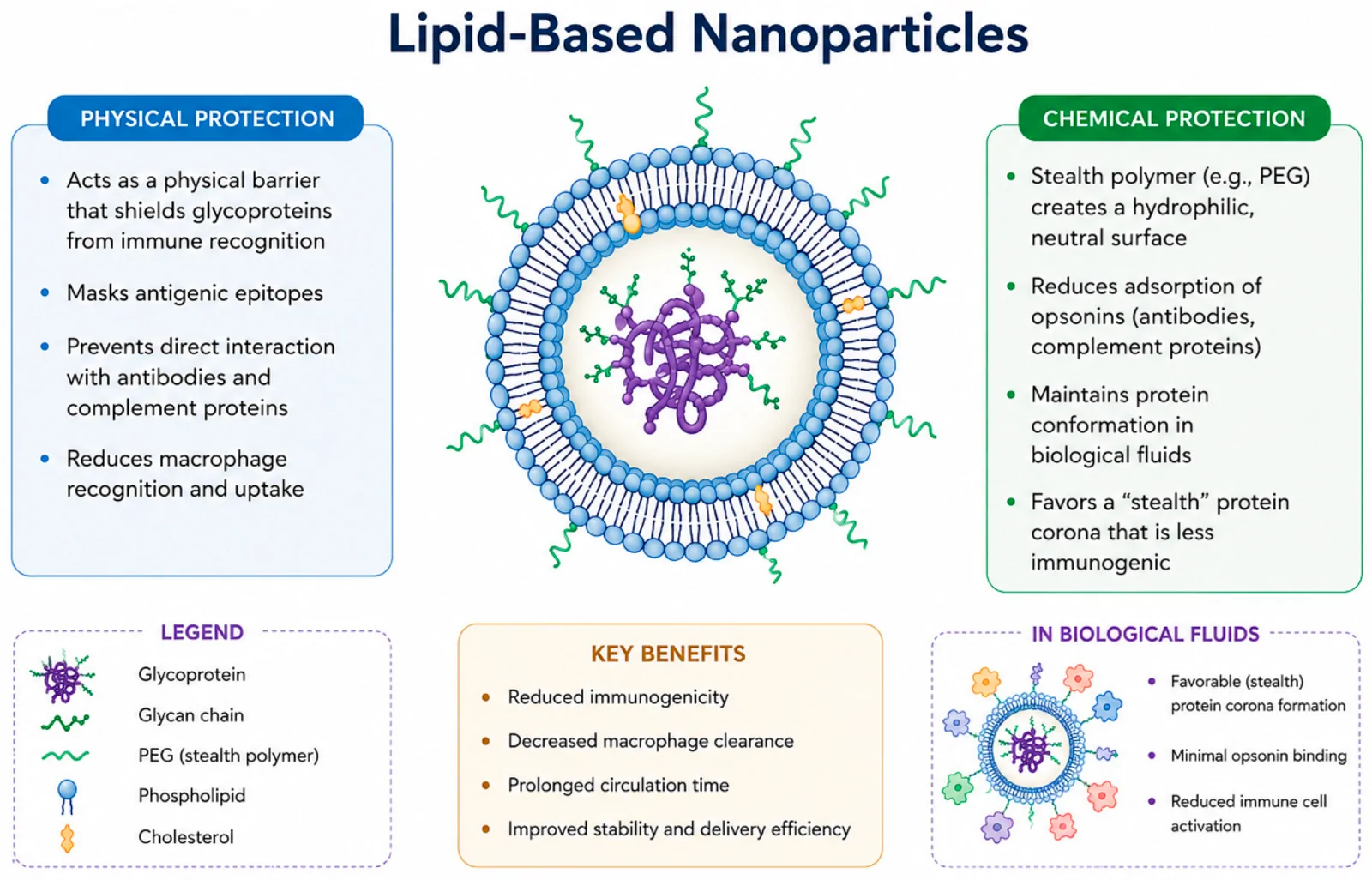
Lipid-based delivery systems of glycoproteins.

Lipid-based nanoparticles represent an innovative and effective approach for delivering glycoproteins, addressing many of the limitations associated with conventional delivery systems, such as poor stability, rapid degradation, and low bioavailability. These nanoparticles protect fragile glycoproteins from enzymatic degradation, chemical and physical denaturation, thereby prolonging their functional lifespan in biological systems (Figure 2). Lipid-based nanoparticles can lower the immunogenicity of glycoproteins and proteins by shielding them from immune system recognition via imparting “stealth” properties that reduce macrophage-mediated uptake and clearance, as well as by influencing the composition of the protein corona, primarily proteins that spontaneously adsorbed onto the surface of nanoparticles when exposed to biological fluids, thereby affecting nanoparticle–biological interactions, biodistribution, and immune responses [77].

Although several non-lipid platforms, including polymeric nanoparticles, hydrogels, dendrimers, extracellular vesicles, microneedles, and implantable delivery systems, have been explored for therapeutic protein delivery, lipid-based carriers offer several distinct advantages for glycoproteins. Their biocompatibility and structural similarity to biological membranes enable efficient encapsulation while preserving the native conformation and biological activity of structurally sensitive glycoproteins [78]. Furthermore, lipid-based systems protect glycoproteins from enzymatic degradation, facilitate cellular uptake through membrane fusion or endocytosis, allow versatile surface modification with polyethylene glycol (PEG) or targeting ligands, and can be engineered for controlled or stimulus-responsive release [79]. Compared with many alternative platforms, lipid-based nanocarriers generally provide a favorable balance between biocompatibility, delivery efficiency, scalability, and clinical translation, as demonstrated by the successful approval of several lipid-based nanomedicines. These characteristics make lipid-based delivery systems particularly attractive for therapeutic glycoproteins that require protection, targeted delivery, and preservation of biological function.

Glycoproteins can exhibit limited apparent solubility due to aggregation, instability, or denaturation under physiological conditions [80]. Lipid-based delivery systems significantly enhance the solubility of glycoproteins by modifying their physicochemical environment and preventing aggregation in aqueous media. Encapsulation within lipid-based nanoparticles, such as liposomes or micelles, creates a hydrated, protective microenvironment that improves dispersion and stability in biological fluids [81]. From a physical standpoint, lipid-based nanoparticles act as carriers that disperse glycoproteins at the nanoscale, reducing intermolecular interactions that lead to aggregation and precipitation. The high surface area and uniform distribution increase apparent solubility and maintain the protein in a stable, bioavailable form [82]. From a chemical viewpoint, surface modification (e.g., PEGylation) of lipid-based nanoparticles introduces a hydrophilic shell that enhances interaction with aqueous environment and reduces hydrophobic interactions between protein molecules [83].

Despite the significant advances achieved with lipid-based nanocarrier systems, several limitations continue to restrict their widespread clinical translation. Encapsulation of large hydrophilic glycoproteins may be associated with limited loading efficiency and premature drug leakage, while lipid oxidation, hydrolysis, and physical instability can compromise formulation integrity during storage [84]. In vivo, rapid recognition and clearance by the mononuclear phagocyte system may reduce circulation time and target-site accumulation, whereas repeated administration of PEGylated formulations may elicit anti-PEG antibodies and accelerated blood clearance in susceptible individuals [85]. Furthermore, the complexity of large-scale manufacturing, sterilization, batch-to-batch reproducibility, and long-term storage stability presents additional challenges for commercialization. These limitations highlight the need for continued advances in lipid engineering, formulation optimization, scalable manufacturing technologies, and quality control to improve the stability, safety, and clinical performance of lipid-based glycoprotein delivery systems.

## 5. Lipid-Based Nanocarriers for Glycoprotein Delivery

### 5.1. Design Principles and Formulation Strategies for Lipid-Based Glycoprotein Delivery

The successful formulation of lipid-based nanocarriers for therapeutic glycoproteins requires careful selection of lipid components to preserve protein structure, maximize encapsulation efficiency, facilitate intracellular delivery, and maintain long-term stability. Because glycoproteins possess complex tertiary structures and glycan moieties that are highly sensitive to environmental stress, formulation strategies must minimize protein denaturation while providing protection against enzymatic degradation and premature clearance.

Lipid selection is one of the primary determinants of nanoparticle performance. Phospholipids such as phosphatidylcholine (PC), hydrogenated soy phosphatidylcholine (HSPC), dioleoyl phosphatidylcholine (DOPC), and distearoyl phosphatidylcholine (DSPC) provide the structural framework of lipid-based delivery systems. Saturated phospholipids, such as dipalmitoylphosphatidylcholine (DPPC) and DSPC, generally produce more rigid bilayers with improved stability and slower drug leakage [86], whereas unsaturated phospholipids, such as DOPC, enhance membrane fluidity and facilitate membrane fusion and intracellular uptake [87]. The transition temperature of the phospholipid also influences membrane permeability and release kinetics. Phospholipids with higher temperature of transitions (e.g., HSPC and DPPC) better retained entrapped molecules while in the circulation [88].

Helper lipids are non-primary structural components added to and lipid nanoparticles to improve stability, control membrane fluidity, and assist in intracellular drug or nucleic acid delivery. Dioleoyl phosphatidylethanolamine (DOPE) promotes membrane fusion and facilitates endosomal escape by adopting a non-bilayer hexagonal phase under acidic conditions [89]. Other helper lipids, including phosphatidylethanolamine derivatives and fusogenic lipids, enhance intracellular release by destabilizing endosomal membranes after cellular uptake.

Cholesterol is incorporated into most lipid formulations to regulate membrane fluidity and mechanical strength. By filling gaps between phospholipid molecules, cholesterol decreases membrane permeability, reduces premature leakage of encapsulated glycoproteins, improves serum stability, and prolongs circulation time [90]. The optimal cholesterol concentration depends on the phospholipid composition and desired release profile, as excessive cholesterol (more than 50%) may reduce encapsulation efficiency or impair controlled release [91].

PEGylation remains one of the most widely used surface engineering strategies for lipid nanoparticles. PEG-conjugated lipids such as DSPE-PEG2000 generate a hydrophilic steric barrier around the nanoparticle, reducing opsonization, complement activation, and macrophage-mediated clearance by the mononuclear phagocyte system. This prolongs systemic circulation and enhances passive accumulation at disease sites. However, repeated administration of PEGylated nanoparticles may induce anti-PEG antibodies and accelerated blood clearance, highlighting the need for alternative stealth polymers in future formulations [92].

Ionizable lipids such as 1,2-Dioleoyl-3-dimethylammonium propane (DODAP) have become key components of modern lipid nanoparticles because they enable efficient encapsulation of biologics while minimizing systemic toxicity. These lipids remain largely neutral at physiological pH, reducing nonspecific interactions with serum proteins, but become positively charged within the acidic endosomal environment. Protonation promotes electrostatic interaction with endosomal membranes, facilitating membrane destabilization, endosomal escape, and cytoplasmic delivery of therapeutic cargo [93]. Although ionizable lipids have been primarily developed for nucleic acid delivery, their membrane-disruptive properties may also improve intracellular delivery of selected glycoproteins requiring cytosolic or intracellular targets [94].

Surface functionalization further enhances delivery specificity through the conjugation of targeting ligands, including monoclonal antibodies, antibody fragments, peptides, aptamers, carbohydrates, folic acid, transferrin, and receptor-specific ligands [95]. These modifications promote receptor-mediated endocytosis, increase tissue selectivity, reduce off-target accumulation, and improve therapeutic efficacy. The selection of targeting ligands should consider receptor expression, internalization efficiency, and disease-specific biological characteristics. Lipid nanoparticles can also be engineered to preferentially accumulate in specific organs by modifying lipid composition, particle size, surface charge, or targeting ligands. For example, hepatic targeting has been widely achieved using N-acetylgalactosamine (GalNAc) ligands that bind the asialoglycoprotein receptor expressed on hepatocytes [96].

Monoclonal antibodies and antibody fragments provide highly specific targeting toward disease-associated surface antigens. Antibody-functionalized lipid nanoparticles selectively bind receptors such as EGFR and CD44, enabling selective delivery to tumor cells or activated immune cells [97]. However, compared with small-molecule ligands, antibody-mediated targeting generally provides superior specificity but may increase formulation complexity, production costs, and potential immunogenicity.

Encapsulation strategies must preserve the structural integrity and biological activity of sensitive glycoproteins throughout formulation and storage. Passive encapsulation is suitable for water-soluble glycoproteins entrapped within the aqueous core of liposomes, whereas active loading approaches, electrostatic complexation, lipid–protein conjugation, and microfluidic mixing can improve encapsulation efficiency and formulation reproducibility [98]. Process parameters such as hydration conditions, buffer composition, pH, ionic strength, temperature, and mixing rate should be carefully optimized to minimize protein unfolding, aggregation, and loss of biological activity during nanoparticle preparation.

Collectively, these formulation parameters interact to determine encapsulation efficiency, colloidal stability, release kinetics, biodistribution, intracellular trafficking, and therapeutic performance. Therefore, rational formulation design should integrate both the physicochemical properties of the glycoprotein and the functional roles of individual lipid components to develop efficient and clinically translatable lipid-based delivery systems.

### 5.2. Liposomes

Liposomes are spherical vesicles composed of one or more phospholipid bilayers surrounding an aqueous core. Their unique structure allows the encapsulation of both hydrophilic therapeutic agents, which are entrapped within the aqueous core, and hydrophobic drugs, which are incorporated into the lipid bilayer. Since their discovery by Alec Bangham in the 1960s, liposomes have become one of the most extensively studied and clinically successful drug delivery systems. Liposomes serve as effective drug carriers, demonstrating remarkable qualities like shielding the enclosed compounds from physiological breakdown, prolong circulation time, regulating the release of drug molecules, and possessing excellent biocompatibility and safety [99].

**Figure 3:**
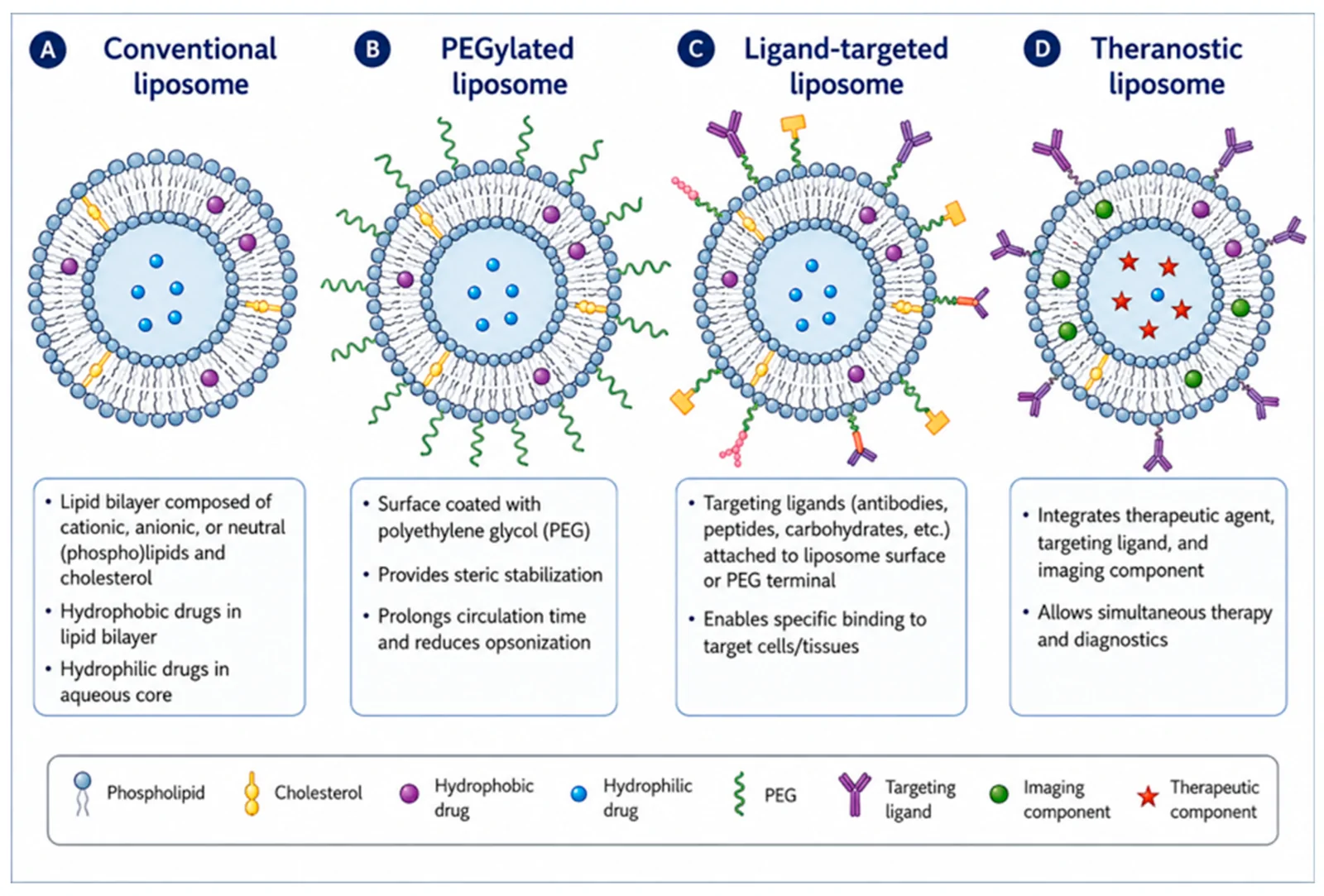
Schematic illustration of the major types of liposomal drug delivery systems, including (**A**) conventional liposomes, (**B**) PEGylated (stealth) liposomes, (**C**) ligand-targeted liposomes, and (**D**) theranostic liposomes.

Liposomes may be classified according to their size and lamellarity into two principal groups: multilamellar vesicles (MLVs), which contain multiple concentric lipid bilayers, and unilamellar vesicles, which possess a single lipid bilayer. Unilamellar vesicles are further categorized as either large unilamellar vesicles (LUVs, >100 nm) or small unilamellar vesicles (SUVs, 30–100 nm) [100]. According to surface properties, liposomes may be conventional, PEGylated (stealth), cationic, or anionic [101]. Functional classifications include passive and active targeting liposomes, immunoliposomes, and stimuli-responsive liposomes (Figure 3).

PEGylated liposomes, commonly referred to as stealth liposomes, are surface-modified liposomal systems containing polyethylene glycol (PEG) chains that provide steric stabilization and reduce recognition by the mononuclear phagocyte system. The hydrophilic PEG coating minimizes protein adsorption and macrophage-mediated uptake, thereby prolonging systemic circulation and improving pharmacokinetic performance [102]. Common PEGylated lipids, such as DSPE-PEG2000 and PEG-DOPE, are incorporated into the liposomal bilayer to enhance stability and biodistribution.

Stimuli-responsive liposomes are advanced lipid-based nanocarriers designed to release their therapeutic payload in response to specific internal or external stimuli, thereby improving site-specific drug delivery and minimizing systemic toxicity [103]. pH-responsive liposomes are the most extensively investigated and commonly composed of dioleoyl phosphatidylethanolamine (DOPE), cholesteryl hemisuccinate (CHEMS), phosphatidylcholine, and PEGylated lipids such as DSPE-PEG2000. Under acidic conditions, protonation of CHEMS destabilizes the liposomal membrane and promotes therapeutic payload release [104].

In addition to pH-responsive systems, several other classes of stimuli-responsive liposomes have been developed. Thermosensitive liposomes are commonly prepared using DPPC, MSPC, DSPC, and DSPE-PEG2000, enabling rapid drug release upon exposure to mild hyperthermia (40–42 °C) [105]. Redox-responsive liposomes typically incorporate disulfide-containing lipids or linkers, including PEG-SS-DOPE, PEG-SS-DSPE, and cystamine-based derivatives, which undergo cleavage in the glutathione-rich intracellular environment [106]. Enzyme-responsive liposomes often contain matrix metalloproteinase-sensitive peptide linkers or phospholipase-sensitive lipid components that facilitate localized drug release at diseased tissues [107]. Light-responsive liposomes utilize photosensitive molecules such as benzoporphyrin derivatives, porphyrins or azobenzene derivatives, compounds that induce membrane destabilization following irradiation [108].

Laronidase-loaded liposomes have been successfully developed for noninvasive intranasal delivery of the glycoprotein enzyme α-L-iduronidase, a recombinant glycoprotein enzyme used in enzyme replacement therapy for Mucopolysaccharidosis Type I (MPS I) [109]. The main characterization of the liposomal formulation, composed of egg yolk lecithin and DOTAP, showed mean a vesicle size of 103.0 ± 3.3 nm, a zeta potential of around + 30.0 ± 2.1 mV, and a mucoadhesion strength of 5.69 ± 0.14 mN. The liposomal formulation enhanced α-L-iduronidase transport to the brain and other poorly accessible organs, highlighting the potential of liposome-mediated nose-to-brain delivery for treating neurological manifestations of lysosomal storage disorders such as MPS I. These findings demonstrate the promise of liposomal carriers in overcoming biological barriers and improving the therapeutic efficacy of glycoprotein-based enzyme replacement therapies.

Arias et al. developed a PEGylated liposomal formulation to improve the pharmacokinetic profile of DNase I, a therapeutic enzyme with a naturally short circulating half-life [110]. Liposomes composed of Egg PC, cholesterol, and DSPE-PEG2000 (50.5:44.5:5 mol%) encapsulated the hydrophobically modified enzyme. The resulting nanoparticles exhibited sizes of approximately 100–130 nm and pharmacokinetic data showed a terminal elimination half-life of 7.1 h, representing a substantial improvement compared with the native enzyme, which exhibited a half-life of only 2.2 h. These results suggest that PEGylated liposomes can reduce rapid clearance and increase circulation time, thereby enhancing the pharmacokinetic performance of therapeutic glycoproteins.

**Figure 4:**
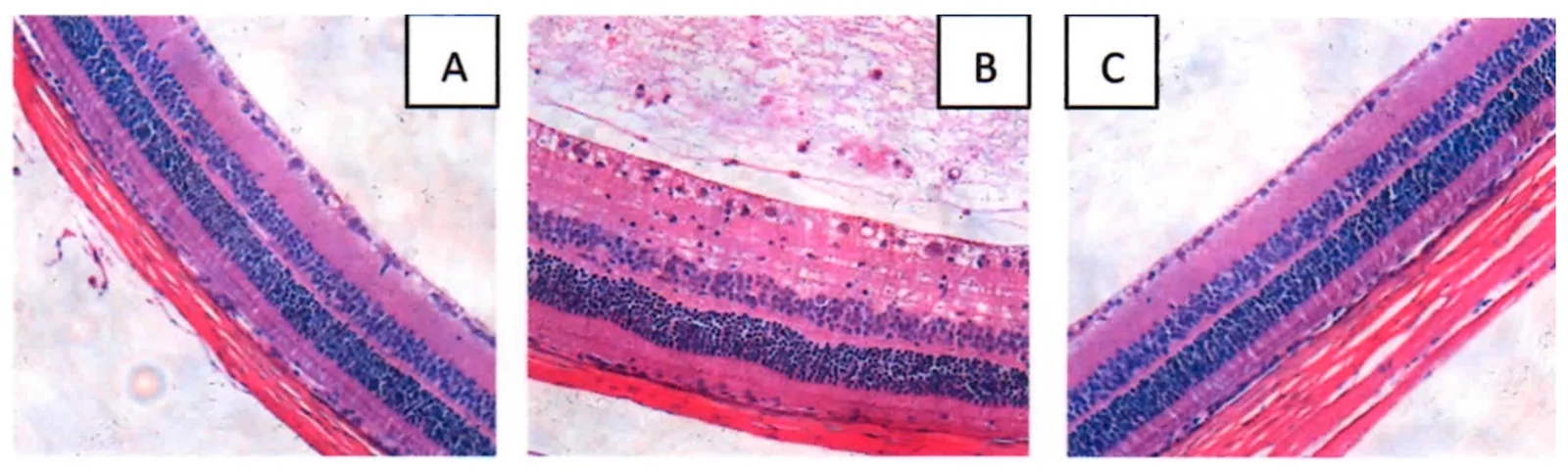
Histological analyses confirmed superior ocular safety of the liposomal formulation, no retinal abnormalities were observed following administration of infliximab-loaded liposomes. H&E-stained retinal sections of normal rat (**A**), normal rats 2 days after intravitreal injection of infliximab (**B**) and infliximab-lip (**C**) (original magnification, ×100). Adapted with permission from Ref. [111].

Zhang et al. developed PEGylated liposomes for the intravitreal delivery of infliximab, a monoclonal anti-TNF-α antibody, for the treatment of experimental autoimmune uveoretinitis [111]. The liposomes were prepared using DOPC, cholesterol, and DSPE-PEG-NH_2_, yielding nanoparticles with a mean particle size of 351.3 ± 58 nm, a zeta potential of −20.8 ± 9.78 mV, and a high encapsulation efficiency of 90.65 ± 2.68%. In vivo, liposomal infliximab significantly reduced ocular inflammation and retinal damage while prolonging vitreous retention of the antibody for up to 45 days, compared with only 14 days for free drug. Furthermore, electroretinography and histological evaluation confirmed superior ocular safety of the liposomal formulation. No retinal abnormalities were observed following administration of infliximab-loaded liposomes, indicating excellent biocompatibility and tolerability within ocular tissues (Figure 4).

Encapsulation of therapeutic cytokines into liposomes has been shown to improve their pharmacokinetic profiles, enhance biological activity, and reduce systemic toxicity. Kedar et al. investigated liposomal delivery of the cytokines granulocyte–macrophage colony-stimulating factor (GM-CSF) and tumor necrosis factor-α (TNF-α) [112]. GM-CSF was encapsulated in stable plurilamellar vesicles composed of phosphatidylcholine (PC), phosphatidylethanolamine (PE), and phosphatidylinositol (PI) at a molar ratio of 22:18:15; 62.5 mg cholesterol; and 0.2 mg butylated hydroxytoluene. TNF-α was incorporated into liposomes composed of egg phosphatidylcholine (EPC), cholesterol, α-tocopherol succinate and butylated hydroxytoluene at a molar ratio of 66:33:0.2:1 mole %. In vivo studies demonstrated enhanced hematopoietic activity of liposomal GM-CSF. Compared with soluble GM-CSF, liposomal GM-CSF produced 2–4-fold increases in peritoneal leukocytes and 3–4-fold increases in spleen colony-forming units (GM-CFU-C). Furthermore, liposomal TNF-α exhibited markedly reduced systemic toxicity, with a 3–7-fold increase in the median lethal dose (LD50) compared with free TNF-α.

Zalba et al. developed cetuximab-conjugated oxaliplatin-loaded liposomes developed as an EGFR-targeted system for colorectal cancer therapy [113]. The liposomes incorporated cetuximab as a targeting glycoprotein against the epidermal growth factor receptor (EGFR)**,** which is frequently overexpressed in colorectal cancer cells. The formulation included: hydrogenated phosphatidylcholine (HSPC or EPC), Cholesterol, PEGylated phospholipid (DSPE-PEG2000), and cetuximab conjugated to the distal end of PEG chains. Surface-conjugated cetuximab facilitated selective recognition of EGFR-overexpressing tumor cells, enhancing cellular uptake, intracellular drug delivery, and antitumor efficacy compared with free oxaliplatin and non-targeted liposomes.

**Figure 5:**
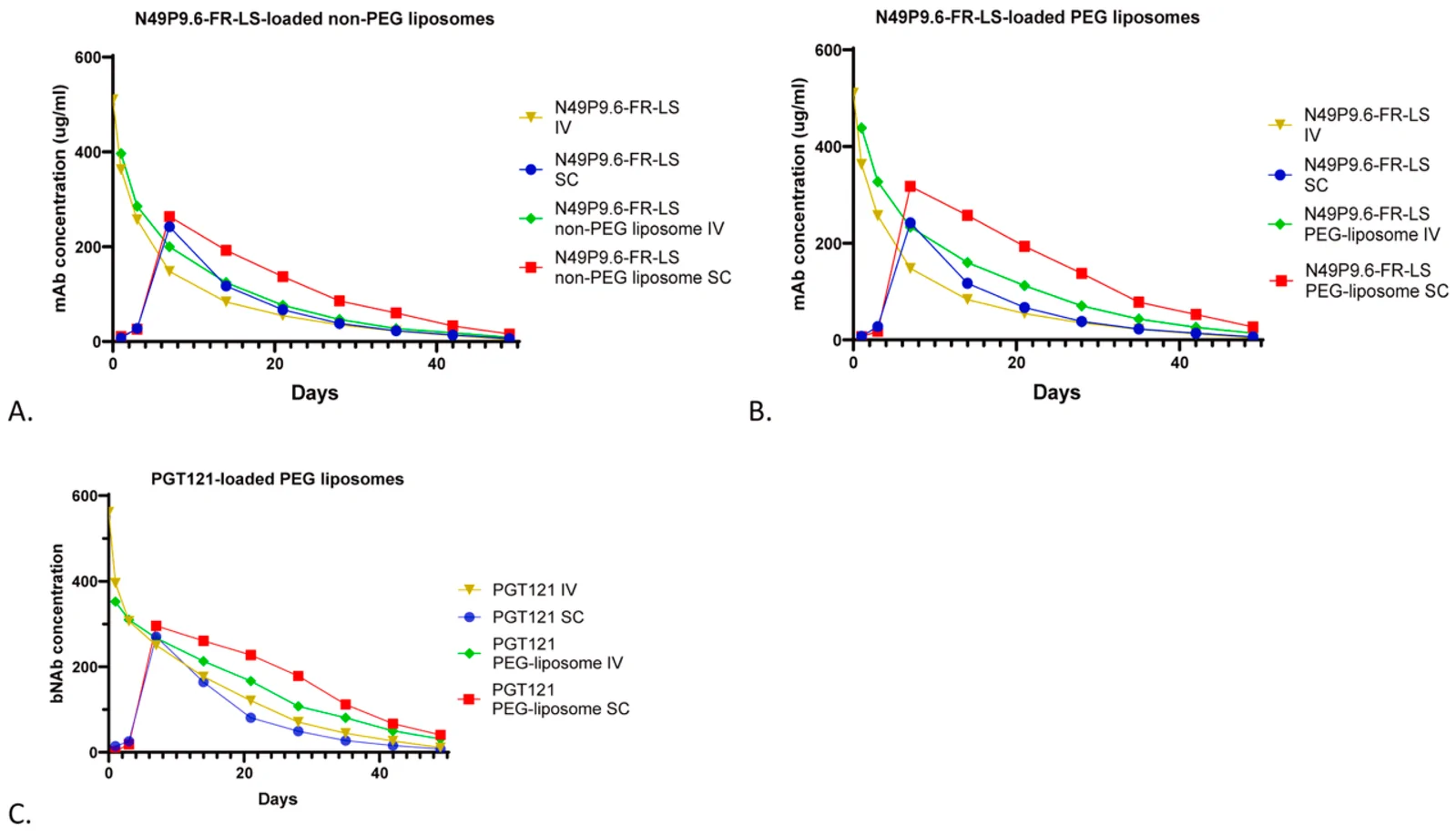
In vivo pharmacokinetics of IV and SC liposomal formulations. (**A**) PK of free mAbs vs. non-PEG liposomes loaded with N49P9.6-FR-LS. (**B**) PK of free mAbs vs. PEG liposomes loaded with N49P9.6-FR-LS. (**C**) PK of free mAbs vs. PEG liposomes loaded with PGT121. When comparing the gold standard (IV injection of free mAb) to subcutaneous injection of PEG liposomal formulation, the subcutaneous liposomal had higher bioavailability than the IV injections, with the N49P9.6-FR-LS-loaded PEG liposomes having 80% higher bioavailability. Adapted with permission from Ref. [115].

PEGylation of liposomes improves their pharmacokinetic behavior by reducing premature leakage, slowing payload release, and extending systemic circulation time through decreased uptake by mononuclear phagocyte system [114]. Karimi et al. developed PEGylated liposomal formulations for the subcutaneous delivery of broadly neutralizing anti-HIV monoclonal antibodies (N49P9.6-FR-LS) [115]. The PEGylated liposomes were composed of HSPC:Chol:DSPG:DSPE-mPEG2000 (in a molar ratio of 50:35:10:5) and exhibited a particle size of approximately 92 nm with an encapsulation efficiency of 61%. In vitro release studies demonstrated that PEGylated liposomes exhibited substantially slower antibody release than their non-PEGylated counterparts, with only 7–9% of the encapsulated antibody released during the first 24 h and prolonged intracellular retention due to slower antibody release. In vivo studies revealed that PEGylated liposomes significantly improved monoclonal antibody pharmacokinetics (Figure 5), yielding up to 113% higher bioavailability and an 81% extension of half-life compared with free subcutaneous antibody administration.

Stimuli-responsive liposomes are advanced drug delivery systems designed to release their payload in response to specific internal or external triggers such as pH, temperature, light, enzymes, redox potential, or ultrasound. These systems provide controlled and site-specific delivery of glycoproteins, thereby improving therapeutic efficacy while minimizing off-target effects [116].

Wang et al. developed a light-responsive liposomal delivery system for the intracellular transport of the anti-Ki-67 monoclonal antibody TuBB-9 [117]. The liposomes were composed of DPPC, DOTAP, cholesterol, and DSPE-PEG2000 at a molar ratio of 15:3:4:3 and exhibited a median particle size of approximately 160 nm. To overcome endosomal entrapment, the authors employed benzoporphyrin derivative monoacid (BPD), which generated reactive oxygen species upon 690 nm irradiation and triggered photochemical internalization of the antibody. Reactive oxygen species disrupted endosomal membranes, releasing the trapped antibody into the cytoplasm. Endosomal escape enabled intracellular antibodies to reach the nucleus and target Ki-67, leading to successful nucleolar localization in approximately 73% of Ki-67-positive cells.

In another study, Miyazaki et al. developed bone marrow-targeted liposomes encapsulating recombinant human erythropoietin (rHuEPO) to enhance the treatment of renal anemia [118]. The liposomes consisted of DPPC, cholesterol, the bone marrow-targeting lipid SA (L-glutamic acid, N-(3-carboxy-1-oxopropyl)-, 1,5-dihexadecyl ester), and PEG-DSPE, with rHuEPO encapsulated in the aqueous core. The resulting liposomal formulation exhibited a particle size of 180 ± 39 nm, a zeta potential of −13.5 ± 1.8 mV, and an encapsulation efficiency of approximately 21.2%. In a cisplatin-induced renal anemia model, liposome-EPO demonstrated superior therapeutic efficacy and enhanced accumulation within bone marrow; approximately 70% of the injected dose was localized to the bone marrow.

Although insulin is not classified as a glycoprotein, it has been widely employed as a model therapeutic protein in the development of lipid-based delivery systems. The physicochemical and biological barriers encountered during insulin delivery, including enzymatic degradation, poor membrane permeability, and limited bioavailability, are also common challenges for many therapeutic glycoproteins. Therefore, advances in insulin delivery can provide valuable insights for the design of glycoprotein-based nanomedicines. Insulin-loaded liposomes encapsulated within calcium-crosslinked alginate hydrogels were developed as an oral delivery system to improve insulin bioavailability [119]. The prepared liposomes, made of phosphatidylcholine (PC) and cholesterol, encapsulating arginine–insulin complexes (AINS), demonstrated particle sizes ranging from 100 to 300 nm, a zeta potential of −22.8 mV, and an encapsulation efficiency of 75.9%. Subsequently, the AINS-loaded liposomes were embedded within a cysteine-modified alginate hydrogel to improve adhesion to the intestinal mucosa and provide enhanced protection against degradation in the gastric environment. In vivo studies demonstrated that oral delivery of AINS-Lip-Gel (40 IU/kg) effectively sustained hypoglycemic activity for over 12 h in mice. The formulation achieved a relative bioavailability of nearly 11% and significantly improved insulin permeation across the intestinal barrier. Targeted lipid-based nanocarriers are functionalized with receptor-recognizing molecules, including antibodies and folic acid, to promote selective interaction with target cells and enhance the precision of drug delivery [120]. Yazdi et al. developed folate-targeted PEGylated liposomes to enhance the oral delivery of insulin [121]. Folic acid was incorporated as a targeting ligand to promote receptor-mediated uptake by intestinal epithelial cells. The liposomes were composed of hydrogenated soy phosphatidylcholine (HSPC), cholesterol, methoxy polyethylene glycol–distearoyl phosphatidylethanolamine (mPEG2000-DSPE), and folic acid-conjugated DSPE-PEG3400 (DSPE-PEG3400-FA). The prepared liposomes exhibited particle sizes ranging from approximately 160 to 270 nm, negative zeta potentials, and insulin encapsulation efficiencies between 60% and 75%. Evaluation of antidiabetic activity and insulin pharmacokinetics in streptozotocin-induced diabetic rats showed that the PEG-Lip-FA 1% formulation produced the most pronounced hypoglycemic effect, with significant reductions in blood glucose levels and increased serum insulin concentrations observed approximately 4 h after oral administration and sustained up to 24 h. These findings highlight the potential of folate-targeted PEGylated liposomes to enhance the oral bioavailability and therapeutic efficacy of glycoprotein therapeutics through receptor-mediated intestinal transport.

### 5.3. Solid Lipid Nanoparticles

**Figure 6:**
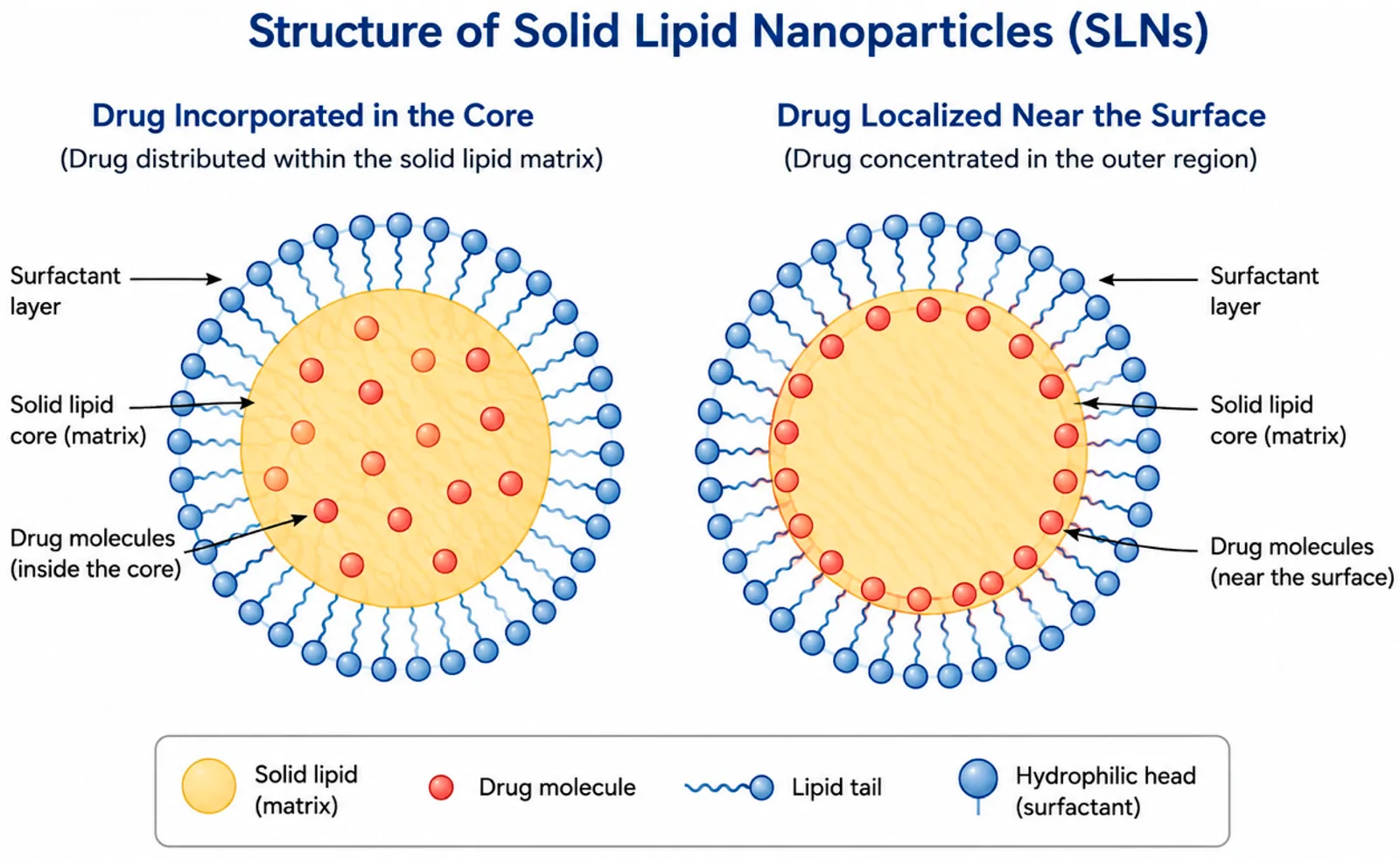
Structural models of solid lipid nanoparticles illustrating different drug incorporation patterns. In the homogeneous matrix model, the drug is distributed throughout the lipid core, whereas in the drug-enriched shell model, the drug is primarily localized near the outer region of the nanoparticle.

Solid Lipid Nanoparticles (SLNs) are submicron-sized colloidal carriers, typically ranging from 50 to 1000 nm in diameter, composed of physiologically compatible lipids that remain solid at both room and body temperatures. Introduced as an alternative to conventional lipid-based delivery systems. SLNs consist of a solid lipid core stabilized by surfactants, within which therapeutic agents can be incorporated. Their solid structure protects encapsulated therapeutic agents from chemical and enzymatic degradation while enabling sustained release and enhanced bioavailability [122]. The lipid matrix of SLNs is commonly prepared using materials such as stearic acid, glyceryl monostearate (GMS), tristearin, tripalmitin, cetyl palmitate, and Compritol^®^ 888 ATO, whereas stabilizers include poloxamer 188, pluronic F68, tween 80, lecithin, and sodium cholate [123]. Depending on the physicochemical properties of the drug, molecules may be distributed homogeneously throughout the lipid matrix or localized near the particle surface (Figure 6). SLNs can be produced using several techniques, including high-pressure homogenization, microemulsion methods, solvent emulsification, ultrasonication, and hot or cold homogenization [124]. SLNs have been extensively investigated for the delivery of peptides, proteins, vaccines, nucleic acids, and monoclonal antibodies. Their advantages include excellent biocompatibility, low toxicity, protection of sensitive biomolecules, controlled drug release, ease of large-scale production, and the ability to incorporate targeting ligands or stimuli-responsive components [125].

Surface functionalization has significantly expanded the capabilities of solid lipid nanoparticles as targeted drug delivery systems. Active targeting can be achieved through the conjugation of ligands such as folic acid, transferrin, hyaluronic acid, and monoclonal antibodies, which facilitate receptor-mediated uptake by target cells. PEGylated lipids, such as DSPE-PEG, are frequently incorporated to enhance colloidal stability, prolong circulation time, and reduce clearance by the reticuloendothelial system. Stimuli-responsive SLNs incorporating pH-, redox-, or enzyme-sensitive moieties have emerged as promising platforms for site-specific drug release. For example, pH-responsive SLNs are designed to exploit the acidic microenvironments found in intracellular endosomal compartments. These systems typically incorporate pH-sensitive materials such as poly(histidine), poly(β-amino esters), or ionizable lipids (DOTAP) that undergo protonation under acidic conditions [126]. Redox-responsive SLNs are designed to exploit the differences in redox potential between the extracellular environment and intracellular compartments. These systems typically incorporate disulfide bonds (-S-S-) within the lipid matrix, surface coating, or linker molecules. Disulfide bonds remain stable during circulation but undergo cleavage in the presence of high intracellular concentrations of glutathione [127].

Schroeder et al. developed remotely activated lipid nanoparticles capable of synthesizing proteins after exposure to light [128]. The nanoparticles were formulated using 1,2-dimyristoyl-sn-glycero-3-phosphocholine (DMPC), a phospholipid that self-assembles into bilayers at physiological temperature. This lipid-based nanoparticle was filled with amino acids, ribosomes, and plasmid caged with a photo-labile DNA cage (DMNPE) for light-triggered activation. The liposomes successfully produced green fluorescent protein, confirming that the encapsulated machinery remained functional and capable of proper protein folding. A Renilla luciferase-encoding DNA template was incorporated into the particles to assess their ability to produce enzymatically functional proteins. UV irradiation at 365 nm uncaged the DNA, and luciferase was produced. Notably, luciferase expression persisted for at least 24 h after local administration in mice.

In another study, Xu et al. developed hemagglutinin-2 (HA2) functionalized solid lipid nanoparticles to enhance the oral delivery of insulin through an endosomal escape mechanism of the nanoparticles [129]. The nanoparticles were composed of soybean lecithin, tripalmitin, stearic acid, sodium cholate, and pluronic F68, with the fusogenic HA2 peptide incorporated either into the aqueous core or lipid shell. The localization of HA2 within the lipid shell (INS HA2-O-SLNs) was more effective than loading HA2 into the aqueous core. The optimized formulation (INS HA2-O-SLNs) exhibited a particle size of approximately 162 nm, a zeta potential of −16.1 mV, and an insulin encapsulation efficiency exceeding 98%. INS HA2-O-SLNs achieved a 2.19-fold increase in transepithelial insulin transport than conventional SLNs and produced superior hypoglycemic activity in diabetic rats after oral administration.

Ferreira et al. developed a targeted lipid-based delivery system composed of cetyl palmitate SLNs loaded with methotrexate (MTX) and surface-functionalized with etanercept for topical psoriasis treatment [130]. The SLNs were prepared using a hot ultrasonication method, in which cetyl palmitate served as the solid lipid matrix and polysorbate 80 acted as the stabilizing surfactant. To achieve targeted delivery, the nanoparticle surface was modified using DSPE-PEG-NH_2_, which enabled the covalent conjugation of etanercept through carbodiimide (EDC)-mediated coupling reactions. The optimized nanoparticles exhibited mean diameters of approximately 356 nm for etanercept-functionalized MTX-loaded SLNs. Polydispersity index values ranged from 0.19 to 0.21, indicating relatively homogeneous particle population. Skin permeation studies using porcine and human skin demonstrated significantly enhanced MTX deposition within the skin when delivered through SLNs compared with free MTX. In healthy and psoriatic human skin, 70–85% of the applied MTX dose accumulated within skin layers after 8 h.

### 5.4. Nanostructured Lipid Carriers

Nanostructured lipid carriers (NLCs) are lipid nanoparticles developed to overcome the limitations of SLNs, particularly their limited drug-loading capacity and potential drug expulsion during storage. NLCs are composed of a mixture of solid lipids and liquid lipids (oils), creating an imperfect lipid matrix with more structural defects that can accommodate larger amounts of therapeutic agents. This unique structure improves drug loading, enhances stability, and minimizes drug leakage compared with SLNs [131]. NLC formulations include glyceryl monostearate, glyceryl behenate (Compritol^®^ 888 ATO), stearic acid, cetyl palmitate, and tripalmitin, while liquid lipids typically include oleic acid, medium-chain triglycerides (Miglyol^®^ 812), isopropyl myristate, and various vegetable oils. Surfactants such as tween 80, poloxamer 188, and lecithin are frequently used to stabilize the nanoparticles [132].

**Figure 7:**
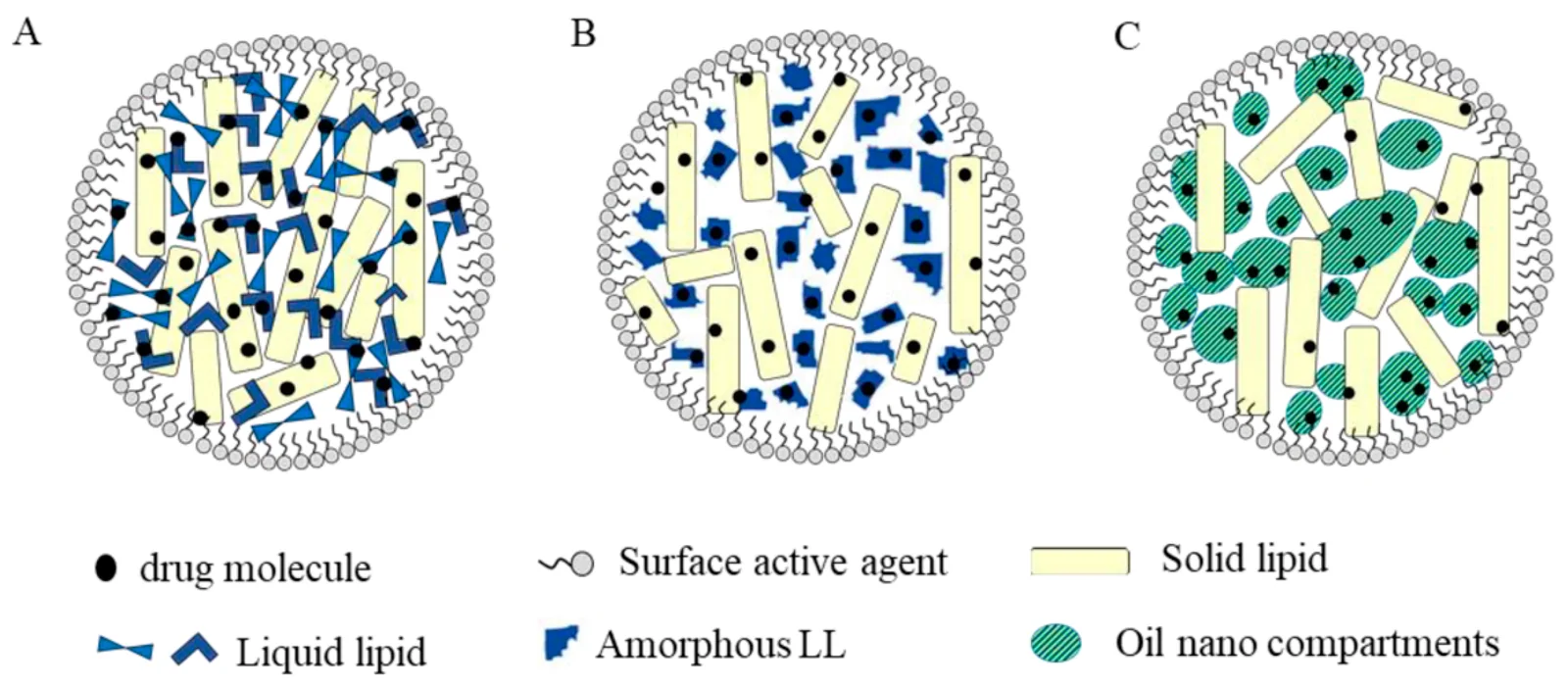
Different types of nanostructured lipid carriers NLCs. (**A**) Imperfect; (**B**) amorphous; and (**C**) oil-in-fat-in-water. Adapted with permission from Ref. [131].

Depending on the composition and arrangement of the lipid matrix, NLCs can be classified into imperfect crystal, amorphous, and multiple-type structures, each offering distinct advantages for drug incorporation and release (Figure 7). Imperfect crystal NLCs are formed by combining solid and liquid lipids with different molecular structures, creating imperfections within the lipid matrix that increase drug-loading capacity. Amorphous NLCs incorporate specific lipids that inhibit crystallization, resulting in an amorphous matrix that minimizes drug expulsion during storage and enhances formulation stability. In contrast, multiple-type NLCs contain small liquid lipid compartments dispersed throughout the solid lipid matrix, enabling high drug loading and sustained drug release [133].

For glycoprotein and protein therapeutics, NLCs provide a protective lipid environment that helps preserve protein integrity and maintain biological activity. Surface modification with PEG, targeting ligands, or mucoadhesive polymers can further improve circulation time, tissue targeting, and cellular uptake. Abdolahpour et al. developed an NLC system functionalized with an anti-EGFRvIII monoclonal antibody, using DSPE-PEG2000-NHS as a linker via a post-insertion method, for the selective delivery of doxorubicin to tumor cells overexpressing EGFRvIII, a tumor-specific mutant epidermal growth factor receptor [134]. The NLCs were composed of stearic acid, oleic acid, lecithin, and poloxamer 188, while DSPE-PEG2000-NHS was employed to conjugate an anti-EGFRvIII monoclonal antibody to the nanoparticle surface. The formulated targeted NLCs exhibited a particle size of 243.2 nm, a zeta potential of −10.5 mV, and an encapsulation efficiency of 67.5%. NLCs demonstrated enhanced cellular uptake and significantly greater cytotoxicity toward EGFRvIII-positive cells, achieving an IC_50_ of 10.12 μg/mL compared with 15.01 μg/mL for non-targeted NLCs.

Liu et al. developed a VEGFR-2-targeted NLC for the delivery of docetaxel using a dual-targeting strategy directed toward both tumor cells and tumor neovasculature [135]. The NLCs were composed of stearic acid (solid lipid), glyceryl monostearate (solid lipid), medium-chain triglycerides (liquid lipid), soya lecithin (emulsifier/lipid matrix), pluronic F68, (surfactant) and DSPE-PEG-NH2, while a monoclonal anti-VEGFR-2 antibody was conjugated to the nanoparticle surface through a PEGylated linker. The targeted NLCs exhibited a particle size of 168.7 nm, a zeta potential of −28.9 mV, an encapsulation efficiency of 98.4%, and a drug loading capacity of 5.55%. In a B16 melanoma mouse model, the antibody-functionalized NLCs demonstrated superior antitumor efficacy compared with non-targeted formulations, owing to increased accumulation within both tumor tissue and tumor-associated vasculature.

Beh et al. prepared erythropoietin (EPO)-conjugated tamoxifen-loaded NLCs for targeted breast cancer therapy by exploiting the overexpression of erythropoietin receptors on tumor cells [136]. The NLCs were composed of hydrogenated palm oil, olive oil, lecithin (Lipoid S100), polysorbate 80, and tamoxifen, while the glycoprotein erythropoietin was adsorbed onto the nanoparticle surface to serve as a targeting ligand. The resulting NLCs exhibited a particle size of 55.4 nm, a zeta potential of −1.58 mV, and an EPO association efficiency of 55.4% at physiological conditions. EPO-tamoxifen-loaded NLCs were approximately 10-fold more cytotoxic toward breast cancer cells than toward normal fibroblasts, indicating improved selectivity and reduced toxicity.

Shahzadi et al. investigated the influence of surface decoration on the performance of NLCs for the oral delivery of therapeutic proteins using insulin, which can be used as a model for glycoprotein delivery owing to the similarities between the delivery barriers encountered by insulin and those associated with many therapeutic glycoproteins [137]. NLC formulations were prepared using stearic acid as the solid lipid and either glyceryl monooleate or oleic acid as liquid lipids, while PEG-ester, PEG-ether, or polyglycerol ester surfactants were employed to modify the nanoparticle surface. The resulting NLCs exhibited particle sizes ranging from approximately 99 to 217 nm and entrapment efficiencies of 58.6–73.0%. PEG-ether NLCs provided the highest protection against pepsin- and pancreatin-mediated insulin degradation, PEG-ether NLCs showed the greatest resistance to lipolysis, releasing only about 10% free fatty acids, compared with over 90% for PEG-ester NLCs. These findings highlight the critical role of surface engineering in enhancing the stability of lipid-based nanocarriers during gastrointestinal transit and suggest that PEG surface modification may represent a promising strategy for improving the oral delivery of proteins and glycoproteins.

### 5.5. Micelles

**Figure 8:**
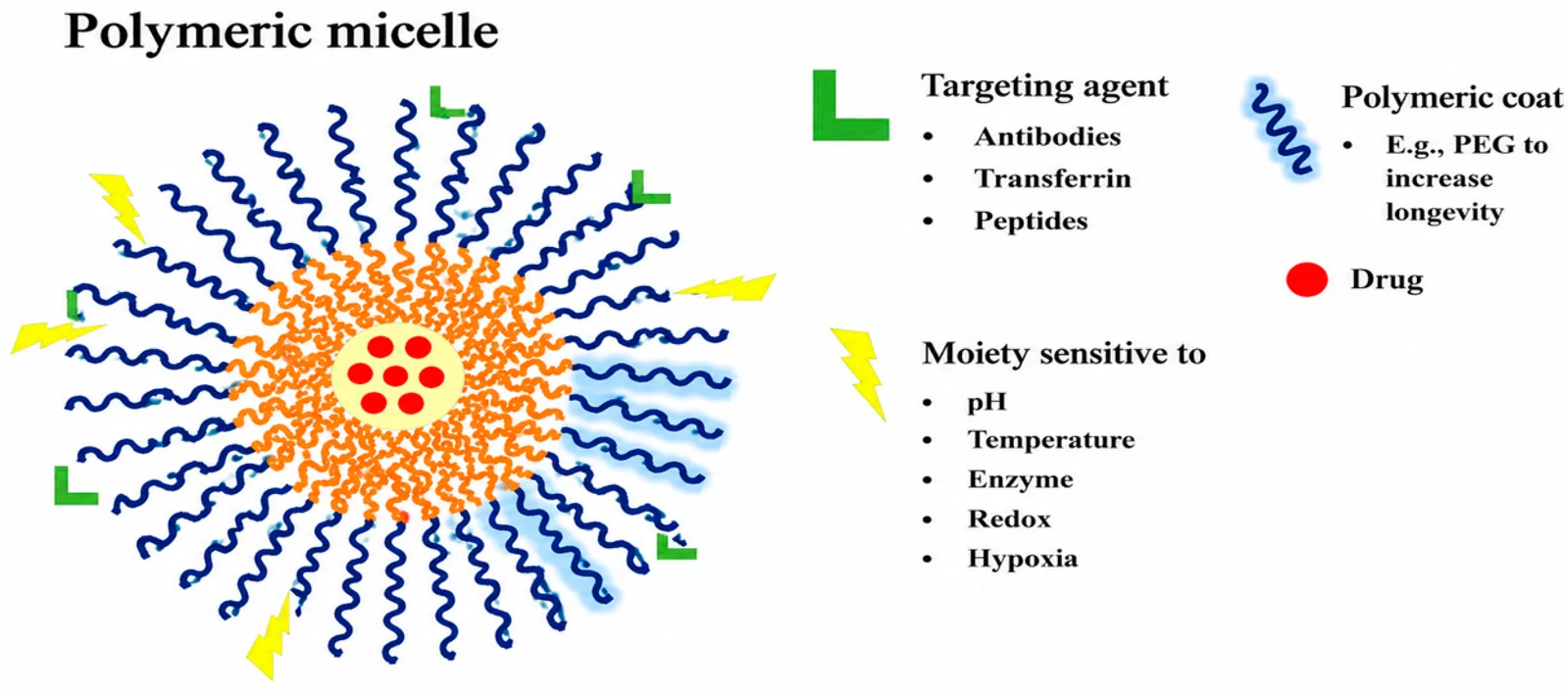
The addition of functional moieties can convert conventional micelles into smart nanocarriers capable of site-specific targeting and controlled cargo release. Adapted with permission from Ref. [139].

Micelles are particularly useful when monoclonal antibodies are used as targeting ligands. Antibody-modified micelles, often referred to as immunomicelles, combine the prolonged circulation and nanoscale size of micelles with the specificity of monoclonal antibodies, resulting in improved therapeutic efficacy [138]. Micelles are nanosized colloidal carriers (10–100 nm) formed by the self-assembly of amphiphilic molecules (surfactants, lipids, or block copolymers) in aqueous media when their concentration exceeds the critical micelle concentration (CMC). Structurally, micelles consist of hydrophobic core, encapsulates poorly water-soluble drugs, and hydrophilic shell (corona), stabilizes the micelle in biological fluids. Micelles formed from amphiphilic block copolymers, such as polyethylene glycol–polylactic acid (PEG–PLA), polyethylene glycol–polycaprolactone (PEG–PCL), polyethylene glycol–phosphatidylethanolamine (PEG–PE), and polyethylene glycol–dioleoyl phosphatidylethanolamine (PEG–DOPE), exhibit greater structural stability, lower critical micelle concentration (CMC), prolonged circulation times, and improved drug retention compared with conventional surfactant-based micelles [139]. By modifying micelles with targeting ligands, stimuli-responsive polymers, or other functional groups, they can be engineered into “smart” delivery systems that enhance targeting efficiency and enable controlled therapeutic release. These advanced micellar platforms offer significant potential for improving the delivery of proteins, glycoproteins, and other biologically active therapeutics (Figure 8).

Lukyanov et al. developed PEG–phosphatidylethanolamine (PEG–PE) micelles as nanocarriers for poorly water-soluble anticancer drugs and further enhanced their tumor-targeting capability through conjugation with the nucleosome-specific monoclonal antibody 2C5 [140]. The system successfully incorporated hydrophobic anticancer agents such as paclitaxel and tamoxifen. In vivo studies using Lewis lung carcinoma-bearing mice demonstrated substantial tumor accumulation through the enhanced permeability and retention (EPR) effect, with PEG5000–PE micelles showing the highest tumor uptake.

Yalamarty et al. developed a 2C5 antibody-targeted dendrimer-based mixed micelle (MDM) system for the co-delivery of doxorubicin and siMDR1 to overcome multidrug resistance in cancer [141]. The formulation consisted of PAMAM-PEG2k-DOPE, PEG5k-DOPE, and 2C5-PEG7k-DOPE conjugates, which self-assembled into nanosized micelles capable of encapsulating doxorubicin within the hydrophobic core while electrostatically complexing siRNA through the cationic PAMAM dendrimer. The monoclonal antibody 2C5 served as a targeting ligand by recognizing tumor-associated nucleosomes exposed on cancer cell surfaces and enhances selective accumulation within tumors. The optimized formulation exhibited particle sizes of approximately 150–165 nm, low polydispersity index, and excellent stability over 20 days. The tumor-targeting monoclonal antibody 2C5 significantly enhanced the cellular association and uptake of doxorubicin in MDA-MB-231 ADR and SKOV-3TR multidrug-resistant cancer cells.

### 5.6. Dual- and Multi-Stimuli-Responsive Lipid-Based Delivery Systems

Dual- and multi-responsive lipid-based delivery systems are advanced nanocarriers designed to respond to two or more internal or external stimuli, including pH, temperature, redox potential, enzymatic activity, light, and ultrasound. These multifunctional systems have shown considerable promise for the delivery of glycoproteins. By integrating several triggering mechanisms, these nanocarriers can enhance cellular uptake, facilitate endosomal escape, improve intracellular delivery, and provide controlled release at the target site [142]. For instance, pH/redox-responsive liposomes might contain pH-sensitive lipids such as DOPE/CHEMS together with disulfide-containing lipids that are cleaved in the glutathione-rich intracellular environment [143]. Likewise, temperature/pH-responsive liposomes can combine thermosensitive lipids such as DPPC with pH-sensitive components to enable drug release under both mild hyperthermia and acidic cellular conditions [144]. These advanced lipid-based nanocarriers represent a promising strategy for overcoming the complex biological barriers associated with glycoprotein delivery and for enhancing the precision of targeted therapeutic interventions.

**Figure 9:**
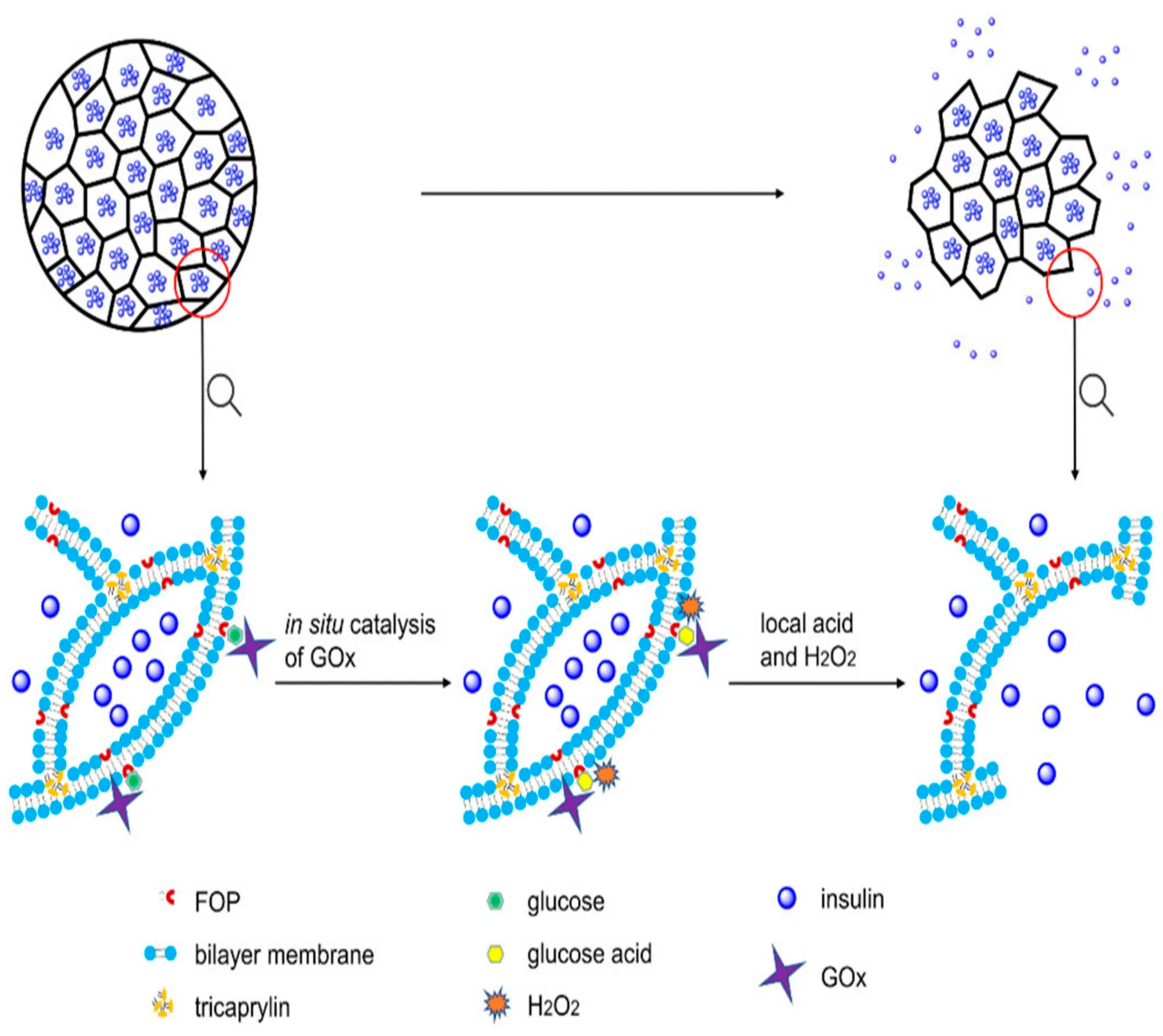
pH-oxidative dual-sensitive glucose-responsive liposomes. Under hyperglycemic conditions, glucose oxidase catalyzed the conversion of glucose into gluconic acid and hydrogen peroxide. The resulting localized acidic and oxidative microenvironment caused protonation of DSPE and lipid peroxidation of the membrane, leading to disruption of the outer liposomal membrane and release of insulin. Adapted with permission from Ref. [145].

While dual- and multi-responsive lipid-based systems for glycoproteins remain relatively underexplored, studies employing insulin as a model protein have demonstrated the feasibility of using multiple endogenous stimuli to regulate protein release. These findings provide a foundation for the future development of stimuli-responsive lipid nanocarriers capable of delivering glycoproteins with enhanced precision, stability, and therapeutic efficacy. Liu et al. developed a glucose-responsive multivesicular liposome system capable of releasing insulin in response to elevated glucose concentrations through a dual pH- and hydrogen peroxide-sensitive mechanism [145]. The lipid membrane components include 1,2-dioleoyl-sn-glycero-3-phosphocholine (DOPC), 1,2-distearoyl-sn-glycero-3-phosphoethanolamine (DSPE), 1,2-dipalmitoyl-sn-glycero-3-phosphoglycerol (DPPG), cholesterol, and tricaprylin. The formulation incorporated insulin as the therapeutic payload, glucose oxidase as the glucose-sensing enzyme, catalase to decompose hydrogen peroxide and regenerate oxygen, and (3-Fluoro-4-((octyloxy)carbonyl)phenyl)boronic acid (FOP), a phenylboronic acid derivative acting as a glucose-binding moiety. Under hyperglycemic conditions, glucose oxidase catalyzed the conversion of glucose into gluconic acid and hydrogen peroxide. The resulting localized acidic and oxidative microenvironment induced protonation of DSPE and promoted lipid peroxidation of the membrane, leading to destabilization of the outer liposomal membrane and release of insulin (Figure 9). The in vivo efficacy of the glucose-responsive liposomes was evaluated using a streptozotocin-induced type 1 diabetic rat model. The formulation effectively regulated blood glucose levels and maintained normoglycemia through self-regulated insulin release.

To provide a practical summary of the concepts discussed throughout this review, Table 7 highlights representative lipid-based formulations for therapeutic glycoprotein delivery, including their composition and the key improvements achieved relative to conventional formulations. The summarized examples illustrate how rational selection of lipid components and nanocarrier systems can address major pharmaceutical challenges, such as poor stability, enzymatic degradation, limited bioavailability, rapid clearance, and insufficient target specificity.

## 6. Concluding Remarks: Challenges and Future Perspectives

Lipid-based nanocarriers have emerged as versatile and highly promising platforms for the delivery of glycoproteins. Lipid-based systems, including liposomes, solid lipid nanoparticles (SLNs), nanostructured lipid carriers (NLCs), lipid nanoparticles (LNPs), and micelles, offer significant advantages by protecting glycoproteins from degradation, enhancing pharmacokinetic profiles, improving biodistribution, and enabling controlled release. Furthermore, surface modifications such as PEGylation, ligand conjugation, and stimuli-responsive functionalities have enhanced targeting specificity and intracellular delivery while reducing immunogenicity and systemic toxicity. The successful development of several clinically approved lipid-based products highlights the translational potential of these nanocarriers and underscores their growing importance in delivering of therapeutic glycoproteins, proteins, enzymes, cytokines, and monoclonal antibodies.

Despite substantial progress, several challenges remain before lipid-based glycoprotein delivery systems can achieve their full clinical potential. Future research should focus on improving glycoprotein loading efficiency while preserving structural integrity and biological activity throughout formulation, storage, and administration. Future research should focus on optimizing the relationship between pharmacokinetics and stimulus-triggered cargo release to achieve precise delivery of glycoprotein therapeutics. For intracellular glycoprotein delivery, nanocarriers must possess sufficiently prolonged circulation and cellular residence times to allow activation by intracellular stimuli, such as acidic endosomal pH or elevated redox potential, thereby facilitating efficient cargo release and maximizing therapeutic efficacy.

Future advances in lipid-based delivery systems are likely to involve the development of stimuli-responsive formulations based on pH, redox potential, enzymes, and temperature. In addition, exploring novel triggers associated with disease-specific metabolic changes, such as lactate, ATP, or reactive oxygen species levels, may provide new opportunities for achieving highly targeted and controlled therapeutic delivery. Despite the growing interest in stimuli-responsive lipid-based nanocarriers, most dual- and multi-responsive systems have been developed for small molecules, nucleic acids, and model proteins such as insulin. Future studies should expand these strategies to therapeutic glycoproteins.

Despite promising preclinical results, the clinical translation of lipid-based delivery systems remains constrained by the complexity of formulation design, manufacturing processes, and large-scale production. Future efforts should focus on developing formulations that combine structural simplicity with high therapeutic performance, as simpler architectures are generally easier to manufacture, characterize, reproduce, and scale up under regulatory requirements.

Future advances in lipid-based glycoprotein delivery are expected to be driven by the integration of artificial intelligence (AI), machine learning (ML), and digital technologies into formulation development. AI- and ML-assisted approaches can accelerate lipid selection, optimize formulation composition, predict critical quality attributes, and model the relationships between formulation variables and biological performance, thereby reducing experimental workload and development time. The emergence of digital twins, virtual models that simulate manufacturing processes and product performance, offers additional opportunities for real-time process optimization, quality control, and scale-up. Furthermore, advances in precision nanomedicine are expected to enable personalized lipid-based formulations tailored to individual patient characteristics, disease biomarkers, and therapeutic responses, thereby improving treatment efficacy while minimizing adverse effects. Continued innovation in next-generation lipid engineering, including the development of biodegradable, stimuli-responsive, and biomimetic lipids, together with AI-driven formulation strategies, is anticipated to further enhance the safety, targeting capability, and clinical translation of therapeutic glycoprotein delivery systems.

## Figures and Tables

**Table 1:** Overview of currently FDA-approved therapeutic glycoproteins.

Agent	Marketing Company	Therapeutic Use
Monoclonal antibody		
Imaavy (Nipocalimab)	Johnson & Johnson	Generalized myasthenia gravis
Enflonsia (Clesrovimab)	Merck	Prevention of RSV in infants
Yartemlea (Narsoplimab)	Omeros	Transplant-associated thrombotic microangiopathy
Gamifant (Emapalumab)	Sobi	Macrophage activation syndrome
Prolia (Denosumab)	Amgen	Osteoporosis
Rituxan (Rituximab)	Genentech Inc., Biogen Idec	B-cell non-Hodgkin lymphoma, chronic lymphocytic leukemia, rheumatoid arthritis
Simponi (Golimumab)	Centocor Ortho Biotech Inc., Merck & Co.	Rheumatoid arthritis, psoriatic arthritis, ankylosing spondylitis, and ulcerative colitis.
Stelara (Ustekinumab)	Centocor Ortho Biotech Inc.	Plaque psoriasis, psoriatic arthritis, Crohn’s disease, and ulcerative colitis.
Vectibix (Panitumumab)	Amgen	EGFR-expressing metastatic colorectal cancer
Humira (Adalimumab)	Abbott Laboratories	Autoimmune inflammatory diseases, including rheumatoid arthritis, psoriatic arthritis, Crohn’s disease, ulcerative colitis
Xolair (Omalizumab)	Genentech Inc., Novartis, Tanox	Moderate-to-severe allergic asthma
Yervoy (Ipilimumab)	BMS	Melanoma
Zevalin (Ibritumomab tiuxetan)	Biogen Idec., Bayer Schering Pharma AG	Relapsed or refractory low-grade or follicular B-cell non-Hodgkin lymphoma.
Bexxar (Tositumomab-I131)	GlaxoSmithKline	CD20-positive follicular non-Hodgkin lymphoma.
Actemra (Tocilizumab)	Genentech Inc., Hoffmann-La Roche Ltd.	Rheumatoid arthritis
Avastin (Bevacizumab)	Genentech Inc., Hoffmann-La Roche Ltd.	Several cancers, including colorectal, lung, renal cell, cervical, ovarian, and glioblastoma indications.
Campath/Mabcampath (Alemtuzumab)	Genzyme Corp.	B-cell chronic lymphocytic leukemia
Herceptin (Trastuzumab)	Genentech Inc., Hoffmann-La Roche Ltd.	HER2-positive breast cancer
Ilaris (Canakinumab)	Novartis Pharmaceuticals	Cryopyrin-associated periodic syndromes
Remicade (Infliximab)	Centocor Ortho Biotech Inc.	Rheumatoid arthritis, Crohn’s disease, ulcerative colitis, ankylosing spondylitis, psoriatic arthritis, and plaque psoriasis.
Reopro (Abciximab)	Centocor Ortho Biotech Inc., Eli Lilly & Co.	Antiplatelet therapy during percutaneous coronary intervention.
Simulect (Basiliximab)	Novartis Pharmaceuticals Corp.	Prevention of acute kidney-transplant rejection.
Zenapax (Daclizumab)	Hoffmann-La Roche Ltd., PDL BioPharma	Prevention of acute kidney-transplant rejection
Synagis (Palivizumab)	Abbott Labs, MedImmune Inc.	Prevention of serious respiratory syncytial virus disease in high-risk pediatric patients.
Tysabri (Natalizumab)	Élan Pharmaceuticals, Biogen Idec	Relapsing forms of multiple sclerosis and Crohn’s disease.
Erbitux (Cetuximab)	ImClone Systems, BMS	Head and neck squamous cell carcinoma and KRAS/RAS wild-type colorectal cancer.
Orthoclone Okt3 (Muromonab-CD3)	Centocor Ortho Biotech Inc.	Treatment or prevention of acute organ-transplant rejection.
Soliris (Eculizumab)	Alexion Pharmaceuticals	Paroxysmal nocturnal hemoglobinuria, atypical hemolytic uremic
Arzerra (Ofatumumab)	GlaxoSmithKline	Chronic lymphocytic leukemia
Benlysta (Belimumab)	Human Genome Sciences Inc.	Systemic lupus erythematosus and lupus nephritis.
Mylotarg (Gemtuzumab ozogamicin)	Wyeth Pharmaceuticals	CD33-positive acute myeloid leukemia.
Fc-Fusion Proteins		
Eylea (Aflibercept)	Bayer	Retinal diseases
Enbrel (Etanercept)	Amgen, Wyeth Pharmaceutical	Rheumatoid arthritis, psoriatic arthritis, ankylosing spondylitis, plaque psoriasis
Arcalyst (Rilonacept)	Regeneron Pharmaceutical Inc.	Cryopyrin-associated periodic syndromes and recurrent pericarditis.
Amevive (Alefacept)	Astellas Pharma Inc.	Moderate-to-severe chronic plaque psoriasis.
Orencia (Abatacept)	BMS	Rheumatoid arthritis, psoriatic arthritis, and juvenile idiopathic arthritis.
Nulojix (Belatacept)	BMS	Prevention of kidney-transplant rejection.
Hormone		
Thyrogen (Thyrotropin α)	Genzyme Corp.	Diagnostic testing and radioiodine ablation support in differentiated thyroid cancer.
Serostim/Saizen/Zorbtive (Somatropin)	EMD Serono, Inc.	Growth hormone deficiency
Luveris (LH)	EMD Serono, Inc.	Infertility treatment in women with luteinizing hormone deficiency
OP-1 Putty (Osteogenic Protein-1)	Stryker Biotech	Bone regeneration/orthopedic
Follistim (Follitropin-β)	Merck & Co.	Infertility treatment
Gonal-F (Follitropin alfa)	EMD Serono, Inc.	Infertility treatment
Ovidrel (Choriogonadotropin α)	EMD Serono, Inc.	Triggering final follicular maturation and ovulation in assisted reproduction/infertility treatment.
Emdogain (Tooth enamel proteins)	Straumann USA	Periodontal tissue regeneration.
Cytokine		
NeoRecormon (Epoetin β)	Hoffmann-La Roche Ltd.	Anemia associated with chronic kidney disease, chemotherapy
Avonex (Interferon β-1a)	Biogen Idec, Inc.	Relapsing forms of multiple sclerosis.
Rebif (Interferon β-1a)	Pfizer, EMD Serono	Relapsing forms of multiple sclerosis.
Aranesp (Darbepoetin α)	Amgen	Anemia associated with chronic kidney disease or chemotherapy
Procrit/Epogen (Epoetin α)	Amgen, Centocor Ortho Biotech Inc.	Anemia associated with chronic kidney disease, zidovudine therapy,
Clotting factor		
Helixate FS (Factor VIII)	ZLB Behring	Treatment and prevention of bleeding in hemophilia A.
Xigris (Drotrecogin α)	Eli Lilly & Co.	Treatment and prevention of bleeding in hemophilia A.
Kogenate FS (Factor VIII)	Bayer Schering Corp.	Bleeding episodes and perioperative management in hemophilia with inhibitors and selected factor VII deficiency/Glanzmann thrombasthenia settings.
Advate (Antihemophilic Factor)	Baxter International Inc.	Treatment and prevention of bleeding in hemophilia B.
BeneFIX (Factor IX)	Wyeth Pharmaceuticals	Treatment and prevention of bleeding in hemophilia A.
NovoSeven (Factor VIIa)	Novo Nordisk	Treatment and prevention of bleeding in hemophilia A.
ReFacto (Antihemophilic Factor)	Wyeth Pharmaceuticals	Treatment and prevention of bleeding in hemophilia A.
Xyntha (Factor VIII)	Wyeth Pharmaceuticals	Treatment and prevention of bleeding in hemophilia A.
Enzyme inhibitor		
ATryn (Antithrombin/ATIII)	GTC Biotherapeutics	Prevention of thromboembolic events in hereditary antithrombin
Enzyme		
Myozyme/Lumizyme (Alglucosidase alfa)	Genzyme Corp.	Enzyme replacement therapy for Pompe disease.
Naglazyme (GalNAc 4-sulfatase)	BioMarin Pharmaceutical Inc.	Enzyme replacement therapy for mucopolysaccharidosis VI.
Pulmozyme (Human DNase)	Genentech Inc., Hoffmann-La Roche Ltd.	Improvement of pulmonary function in cystic fibrosis by reducing mucus viscosity.
Elaprase (Idursulfase)	Shire Pharmaceuticals	Enzyme replacement therapy for mucopolysaccharidosis II (Hunter syndrome).
Activase/Cathflo/Actilyse (Alteplase)	Genentech Inc., Boehringer Ingelheim	Acute ischemic stroke, acute myocardial infarction, pulmonary embolism, and catheter clearance depending on product.
Aldurazyme (Laronidase)	Genzyme Corp.	Enzyme replacement therapy for mucopolysaccharidosis I.
Cerezyme (Imiglucerase)	Genzyme Corp.	Enzyme replacement therapy for Gaucher disease type 1.
Fabrazyme (Agalsidase-β)	Genzyme Corp.	Enzyme replacement therapy for Fabry disease.
Hylenex/Cumulase (Hyaluronidase)	MediCult/Halozyme/Baxter	Facilitation of subcutaneous fluid/drug dispersion
Creon (Pancrelipase)	Abbott Products, Inc.	Pancreatic enzyme replacement therapy for exocrine pancreatic insufficiency.
Pancreaze (Pancrelipase)	Ortho McNeil Janssen	Pancreatic enzyme replacement therapy for exocrine pancreatic insufficiency.
Vitrase (Hyaluronidase)	ISTA Pharmaceuticals	Facilitates dispersion/absorption of injected drugs/fluids and contrast agent resorption.
TNKase (Tenecteplase)	Genentech Inc.	Acute myocardial infarction; also used in selected thrombolysis protocols.
Amphadase (Hyaluronidase)	Amphastar Pharmaceuticals	Facilitates absorption and dispersion of injected drugs/fluids and contrast agent resorption.
Antisera		
Thymoglobulin (Anti-thymocyte globulin)	Genzyme Corp.	Prevention or treatment of kidney transplant rejection
Atgam (Anti-thymocyte globulin)	Pfizer, Inc.	Management of renal transplant rejection and treatment of aplastic
DigiFab (Digoxin Immune Fab)	Savage Laboratories	Treatment of life-threatening digoxin or digitoxin toxicity.
CroFab (Crotalidae Polyvalent Immune Fab)	Savage Laboratories	Treatment of North American crotalid snake envenomation.

**Table 2:** Chemical stability challenges in glycoprotein delivery.

Challenge	Mechanism	Impact	Refs.
Oxidation	Reactive oxygen species (ROS) or exposure to oxygen can oxidize amino acid residues, especially methionine, tryptophan, and cysteine, in glycoproteins.	Alters the structural conformation of the glycoprotein. Reduces biological activity and binding affinity. Increases susceptibility to aggregation.	[23,24]
Deamidation	The amide groups of asparagine and glutamine residues in glycoproteins can hydrolyze, leading to the formation of aspartic acid or isoaspartic acid.	Changes the charge and conformation of the protein. Affects the glycoprotein’s biological activity and stability. Can lead to immunogenic responses due to altered epitopes.	[25]
Hydrolysis	The peptide bonds or glycosidic bonds in glycoproteins can be cleaved by hydrolysis, often accelerated under extreme pH conditions (acidic or basic environments).	Leads to fragmentation of the protein or loss of carbohydrate chains. Results in loss of structural integrity and therapeutic function.	[26,27]
Maillard reaction (Glycation)	Non-enzymatic reaction between the protein‚ amino groups (lysine or arginine residues) and reducing sugars leads to glycation.	Formation of advanced glycation end products (AGEs) can impair biological activity. Alters the glycoprotein physical and chemical properties, including solubility and stability.	[28]
Disulfide bond scrambling	Improper reformation of disulfide bonds between cysteine residues can occur during stress conditions such as manufacturing, storage, or delivery.	Results in misfolding or aggregation. Reduces the biological activity of the glycoprotein.	[29]
Isomerization	Conversion of amino acid residues, such as aspartic acid, to isoaspartic acid occurs through rearrangement.	Alters the structural conformation and functionality of glycoproteins. May reduce receptor binding efficiency or therapeutic potency.	[29]
Aggregation	Chemical modifications like oxidation or deamidation can expose hydrophobic regions, leading to self-association or aggregation of glycoproteins.	Reduces bioavailability and increases immunogenicity. Aggregates may trigger adverse immune reactions.	[30]

**Table 3:** Physical stability challenges in glycoprotein delivery.

Challenge	Description	Impact	Refs.
Irreversible Conformational Changes	Glycoproteins are prone to structural denaturation under environmental stress such as changes in temperature, pH, or ionic strength.	Loss of biological activity and impaired therapeutic outcomes.	[33]
Local and Global Unfolding	Noncovalent nature makes glycoproteins vulnerable to unfolding, exposing hydrophobic regions and destabilizing the molecule.	Can trigger aggregation or degradation, reducing stability.	[34]
Precipitation	Glycoproteins, being colloidal, precipitate under unfavorable conditions like extreme pH, high temperature, or high protein concentrations.	Reduces bioavailability and complicates pharmaceutical formulation.	[35]
Surface Adsorption	Glycoproteins may adsorb onto surfaces of delivery devices or storage containers.	Leads to significant loss of active protein, especially in low-concentration formulations.	[36]
Nonnative Supramolecular Aggregation	Environmental factors can promote irreversible aggregation into nonnative states.	Reduces therapeutic efficacy and may provoke immune responses in vivo.	[33]
Shear and Mechanical Stress	Sensitive to agitation and shear stress during manufacturing or transport, leading to conformational instability.	Results in fragmentation, unfolding, or aggregation, compromising functionality and stability.	[37]

**Table 4:** Enzymatic degradation of glycoproteins.

Category	Enzymes	Mechanism	Impact	Refs.
Enzymatic Degradation of the Protein Backbone	Proteases (Endopeptidases: trypsin, chymotrypsin; Exopeptidases: carboxypeptidase, aminopeptidase).	Proteases recognize specific sequences or structural motifs in the protein backbone and hydrolyze the peptide bonds.	Loss of structural integrity, reduction in biological activity, enhanced clearance from the body.	[39]
Enzymatic Degradation of the Glycan Chains	Glycosidases (Exoglycosidases: β-galactosidase, α-mannosidase; Endoglycosidases: endo-β-N-acetylglucosaminidase).	Glycosidases recognize specific glycosidic linkages and hydrolyze them, removing or degrading critical glycan structures.	Loss of glycan-mediated protection from proteolytic degradation, altered pharmacokinetics, increased immune system exposure.	[40]
Dual Action of Proteases and Glycosidases	Proteases (e.g., cathepsins) and Glycosidases (e.g., hexosaminidases).	Simultaneous degradation of the protein backbone and glycan chains by lysosomal enzymes during cellular recycling.	Accelerated degradation in biological environments, loss of functional activity.	[41]

**Table 5:** Summarizing the factors contributing to glycoprotein immunogenicity.

Factor	Details	Refs.
Protein Component	Peptide epitopes, improper folding, and non-human protein sources trigger immune responses.	[44]
Carbohydrate Component	Unique glycan structures altered glycosylation patterns, and mannose-rich glycans contribute to immunogenicity.	[46]
Aggregates and Impurities	Protein aggregation and process-related impurities, such as host cell proteins and DNA, provoke immune reactions.	[44]
Delivery and Administration	Subcutaneous or intramuscular administration and adjuvants amplify immune responses.	[48]

**Table 6:** Key formulation and development considerations for lipid-based nanocarriers, including critical quality attributes (CQAs), critical material attributes (CMAs), critical process parameters (CPPs), characterization methods, and formulation challenges.

Carrier System	Key CQAs	Key CAMs	Key CPPs	Common Characterization Methods	Main Formulation Challenges	Refs.
Liposomes	Particle size, PDI, zeta potential, encapsulation efficiency	Lipid composition, lipid ratio, drug-to-lipid ratio, lipid purity	Hydration conditions/time, extrusion parameters	DLS, TEM, HPLC, DSC	Drug leakage, oxidation, aggregation	[66,67,68]
Lipid nanoparticles (LNPs)	Particle size, encapsulation efficiency	lipid composition, lipid ratio, ionizable lipid properties	Mixing conditions, flow rate	DLS, Cryo-TEM, HPLC	Scale-up, long-term stability	[69]
Solid lipid nanoparticles (SLPs)	Particle size, crystallinity, drug loading	Lipid type, surfactant type and concentration, drug-to-lipid ratio	Homogenization pressure, cooling rate	DLS, DSC, XRD	Drug expulsion, polymorphic changes	[63]
Nanostructured lipid carriers (NLCs)	Particle size, encapsulation efficiency, lipid matrix stability	Solid/liquid lipid ratio, lipid composition, lipid purity, surfactant type and concentration	Homogenization conditions	DLS, TEM, DSC	Lipid compatibility, reproducibility	[69]
Micelles	Particle size, drug loading, stability	Polymer/lipid composition, polymer/lipid ratio, critical micelle concentration (CMC)	Preparation conditions (e.g., mixing conditions, solvent removal or dialysis conditions)	DLS, TEM	Low stability after dilution, premature release	[69]

**Table 7:** Recent studies presenting different lipid-based nanocarrier for glycoprotein delivery and their key formulation strategies and therapeutic improvements.

Therapeutic Molecule	Therapeutic Area	Formulation Composition	Key Improvements	Refs.
Liposomes				
Recombinant α-L-iduronidase	Mucopolysaccharidosis Type I	Intranasal delivery. Lecithin and DOTAP	The liposomal formulation enhanced α-L-iduronidase transport to the brain	[109]
Deoxyribonuclease I	Cystic fibrosis	Egg PC, cholesterol, and DSPE-PEG2000 (50.5:44.5:5 mol%)	Nanoparticles showed a terminal elimination half-life of 7.1 h, which was remarkably higher than that for native DNase, 2.2 h.	[110]
Infliximab	Uveoretinitis	Intravitreal delivery. DOPC, cholesterol, and DSPE-PEG-NH_2_	Liposomal infliximab significantly reduced ocular inflammation and retinal damage	[111]
Granulocyte-macrophage colony-stimulating factor (GM-CSF)	Colony-stimulating factor	PC, PE, PI, cholesterol, and BHT	Liposomal GM-CSF produced 2–4-fold increases in peritoneal leukocytes and 3–4-fold increases in spleen colony-forming units	[112]
Tumor necrosis factor-α (TNF-α).	Immunostimulator	EPC, cholesterol, α-tocopherol succinate, and BHT	Liposomal TNF-α exhibited markedly reduced systemic toxicity, with a 3–7-fold increase in LD50 compared with free TNF-α.	[112]
Cetuximab	Colorectal cancer	Hydrogenated phosphatidylcholine (HSPC or EPC, cholesterol, PEGylated phospholipid (e.g., DSPE-PEG2000), and cetuximab conjugated to the distal end of PEG chains	Surface-bound cetuximab enabled selective recognition of EGFR-overexpressing tumor cells, enhancing cellular uptake, intracellular drug delivery	[113]
Broadly neutralizing anti-HIV monoclonal antibodies (N49P9.6-FR-LS)	HIV	Subcutaneous administration. HSPC, Chol, DSPG, and DSPE-mPEG2000 (in a molar ratio of 50:35:10:5)	In vivo studies revealed that PEGylated liposomes significantly improved monoclonal antibody pharmacokinetics yielding up to 81% extension of half-life	[115]
Monoclonal antibody TuBB-9	Anti-Ki-67	Light-responsive liposomal delivery system. DPPC, DOTAP, cholesterol, and DSPE-PEG2000 (at a molar ratio of 15:3:4:3)	Light-controlled liposomal delivery system enabled intracellular antibodies to reach the nucleus and target Ki-67, leading to successful nucleolar localization in approximately 73% of Ki-67-positive cells.	[117]
Recombinant human erythropoietin	Renal anemia	DPPC, cholesterol, the bone marrow-targeting lipid SA (L-glutamic acid, N-(3-carboxy-1-oxopropyl)-, 1,5-dihexadecyl ester), and PEG-DSPE	In a cisplatin-induced renal anemia model, liposome- erythropoietin enhanced accumulation within bone marrow, approximately 70% of the injected dose localized to bone marrow.	[118]
Solid lipid nanoparticles				
Luciferase	Gene tracking	Light-responsive SNL. 1,2-dimyristoyl-sn-glycero-3-phosphocholine (DMPC)	UV irradiation at 365 nm uncaged the DNA, and luciferase was produced, with luciferase expression persisting for at least 24 h after local administration in mice.	[128]
Hemagglutinin-2 (HA2)	Diabetes	Soybean lecithin, tripalmitin, stearic acid, sodium cholate, and pluronic F68	Insulin- HA2-O-SLNs achieved 2.19-fold higher transepithelial insulin transport than conventional SLNs and produced superior hypoglycemic activity in diabetic rats after oral administration.	[129]
Etanercept	Psoriasis	Topical psoriasis treatment. Cetyl palmitate, polysorbate 80, and surface modification (DSPE-PEG-NH_2_)	Skin permeation studies using porcine and human skin demonstrated significantly enhanced methotrexate deposition within the skin when delivered through SLNs loaded with MTX and surface-functionalized with etanercept. 70–85% of the applied MTX dose accumulated within skin layers after 8 h.	[130]
Nanostructured lipid carriers				
EGFRvIII monoclonal antibody	Solid tumors (HC2 20d2/c cells)	Stearic acid, oleic acid, lecithin, and poloxamer 188. DSPE-PEG2000-NHS was employed to conjugate an anti-EGFRvIII monoclonal antibody	NLCs showed enhanced cellular uptake and significantly greater cytotoxicity toward EGFRvIII-positive cells, achieving an IC50 of 10.12 μg/mL compared with 15.01 μg/mL for non-targeted NLCs	[134]
VEGFR-2	B16 melanoma	Stearic acid (solid lipid), glyceryl monostearate (solid lipid), medium-chain triglycerides (liquid lipid), soya lecithin (emulsifier/lipid matrix), and pluronic F68	In a B16 melanoma mouse model, the antibody-functionalized NLCs achieved superior tumor inhibition owing to increased accumulation within both tumor tissue and tumor vasculature.	[135]
Erythropoietin-tamoxifen	Breast cancer	Hydrogenated palm oil, olive oil, lecithin (Lipoid S100), polysorbate 80, and tamoxifen	EPO- tamoxifen-loaded NLCs were approximately 10-fold more cytotoxic to cancer cells than to normal fibroblasts, indicating improved selectivity and reduced toxicity.	[136]
Micelles				
Nucleosome-specific monoclonal antibody 2C5	Lung carcinoma	PEG-conjugated micelles. PEG–PE	In vivo studies using Lewis lung carcinoma-bearing mice demonstrated significant tumor accumulation through the enhanced permeability and retention (EPR) effect, with PEG5000–PE micelles showing the highest tumor uptake	[140]
Monoclonal antibody 2C5	Cancer (MDA-MB-231and SKOV-3TR cell lines)	Mixed dendrimer-based micelles. PAMAM-PEG2k-DOPE, PEG5k-DOPE, and 2C5-PEG7k-DOPE	The tumor-targeting monoclonal antibody 2C5 enhanced cellular association and uptake of doxorubicin in MDA-MB-231 ADR and SKOV-3TR multidrug-resistant cancer cells.	[141]
Dual- and multi-stimuli-responsive lipid-based delivery systems				
Insulin *	Diabetes	DOPC, DSPE, DPPG, cholesterol, tricaprylin, glucose oxidase, catalase, and FOP	The formulation effectively regulated blood glucose levels and maintained normoglycemia through self-regulated insulin release.	[145]

* Insulin is frequently used as a model protein for evaluating novel drug delivery systems because it faces challenges similar to those encountered by therapeutic glycoproteins, including enzymatic degradation, structural instability, and low bioavailability. As a result, delivery technologies developed for insulin often serve as a foundation for the design of glycoprotein delivery platforms.

## Data Availability

No new data were created or analyzed in this study. Data sharing is not applicable to this article.

## Abbreviations

BHT	butylated hydroxytoluene
BPD	benzoporphyrin derivative monoacid
CHEMS	cholesteryl hemisuccinate
CMAs	critical material attributes
CMC	critical micelle concentration
CPPs	critical process parameters
CQAs	critical quality attributes
Cryo-TEM	cryogenic transmission electron microscopy
DLS	dynamic light scattering
DMPC	1,2-dimyristoyl-sn-glycero-3-phosphocholine
DOPC	1,2-dioleoyl-sn-glycero-3-phosphocholine
DOPE	1,2-dioleoyl-sn-glycero-3-phosphoethanolamine
DOTAP	1,2-dioleoyl-3-trimethylammonium-propane
DPPC	dipalmitoylphosphatidylcholine
DPPG	1,2-dipalmitoyl-sn-glycero-3-phosphoglycerol
DSC	differential scanning calorimetry
DSPC	1,2-distearoyl-sn-glycero-3-phosphocholine
DSPE	1,2-distearoyl-sn-glycero-3-phosphoethanolamine
DSPG	1,2-distearoyl-sn-glycero-3-phospho-(1′-rac-glycerol), sodium salt
EGFR	epidermal growth factor receptor
EPC	egg phosphatidylcholine
EPO	erythropoietin
FOP	(3-Fluoro-4-((octyloxy)carbonyl)phenyl)boronic acid
HA2	hemagglutinin-2
HPLC	high-performance liquid chromatography
HSPC	hydrogenated soy phosphatidylcholine
mPEG2000-DSPE	methoxy polyethylene glycol–distearoyl phosphatidylethanolamine
MPS I	mucopolysaccharidosis Type I
MSPC	1-myristoyl-2-stearoyl-sn-glycero-3-phosphocholine
MTX	methotrexate
NLCs	nanostructured lipid carriers
PAMAM	poly(amidoamine)
PC	phosphatidylcholine
PDI	polydispersity index
PE	phosphatidylethanolamine
PLGA	poly(lactic-co-glycolic acid
PI	phosphatidylinositol
QbD	quality by design
SLNs	solid lipid nanoparticles
TEM	transmission electron microscopy
TNF-α	tumor necrosis factor-α
